# Effective Storage of Electrons in Water by the Formation of Highly Reduced Polyoxometalate Clusters

**DOI:** 10.1021/jacs.1c10584

**Published:** 2022-05-10

**Authors:** Jia-Jia Chen, Laia Vilà-Nadal, Albert Solé-Daura, Greig Chisholm, Takuo Minato, Christoph Busche, Tingting Zhao, Balamurugan Kandasamy, Alexey Y. Ganin, Rachelle M. Smith, Ian Colliard, Jorge J. Carbó, Josep M. Poblet, May Nyman, Leroy Cronin

**Affiliations:** † School of Chemistry, The University of Glasgow, University Avenue, Glasgow G12 8QQ, U.K.; ‡ Department de Química Física i Inorgànica, Universitat Rovira i Virgili, Marcel·lí Domingo 1, Tarragona 43007, Spain; § Department of Chemistry, Oregon State University, Corvallis, Oregon 07331, United States

## Abstract

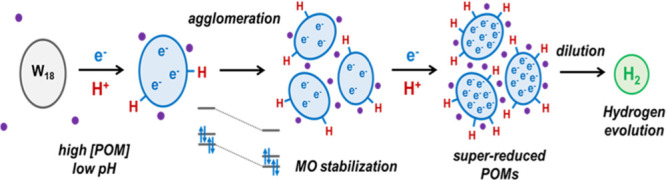

Aqueous solutions of polyoxometalates (POMs) have been shown to have potential as high-capacity energy storage materials due to their potential for multi-electron redox processes, yet the mechanism of reduction and practical limits are currently unknown. Herein, we explore the mechanism of multi-electron redox processes that allow the highly reduced POM clusters of the form {MO_3_}_*y*_ to absorb *y* electrons in aqueous solution, focusing mechanistically on the Wells–Dawson structure X_6_[P_2_W_18_O_62_], which comprises 18 metal centers and can uptake up to 18 electrons reversibly (*y* = 18) per cluster in aqueous solution when the countercations are *lithium*. This unconventional redox activity is rationalized by density functional theory, molecular dynamics simulations, UV–vis, electron paramagnetic resonance spectroscopy, and small-angle X-ray scattering spectra. These data point to a new phenomenon showing that cluster protonation and aggregation allow the formation of highly electron-rich meta-stable systems in aqueous solution, which produce H_2_ when the solution is diluted. Finally, we show that this understanding is transferrable to other salts of [P_5_W_30_O_110_]^15–^ and [P_8_W_48_O_184_]^40–^ anions, which can be charged to 23 and 27 electrons per cluster, respectively.

## Introduction

Molecular metal oxides or polyoxometalates (POMs) are primarily constituted of early-transition-metal elements Mo and W in their highest oxidation states, and so they are susceptible to reduction. Highly reduced POMs have been studied electrochemically since the mid-1970s,^1^ and their high reducibility is still one of the most interesting properties. This rather unusual ability of POMs to accept multiple electrons without losing their structural integrity^2^ inspired terms such as “electron reservoir”^3^ or “electron sponge”.^4^ Over the years, there have been attempts to explain this behavior theoretically. Irle et al. described the electronic structures of the Keggin-type [PMo_12_O_40_]^3–^ heteropolymolybdate and its super-reduced state ([PMo_12_O_40_]^27–^) as well as those of the tungsten analogues,^5^ concluding that the super-reduced POM can be viewed as a “semiporous molecular capacitor” where the formation of the Mo–Mo bond may occur after reduction between 12 and 14 electrons. In the 1960s, Pope started a systematic study finding that the reduction potentials of Keggin anions,^6,7^ with the formula [XW_12_O_40_]^*n*−^ X = P, Ge, Si, and As, among many others, linearly depend on the molecular charge *n.*(8) While this is just an idealized depiction, this empirical rule is able to explain the trend for *n* = 3–7. The linear dependence on molecular charge is also followed by isostructural derivatives of the Keggin anion [XMW_11_O_40_]^*n*−^ with M = V^V^ or Mo^VI^, with the reduction potentials shifted with respect to their homologous 12-tungstates.^9,10^ Several studies over the past decades proved that the electronic structure, and consequently the electrochemistry of POMs, is naturally dependent on the molecular charge,^11^ and more recently, the importance of the countercation^12^ and the environment have been reported.^13−15^ Cyclic-voltammogram experiments provided valuable information on the redox properties of a given species.^16^ In 2018, we reported that lithium salts of the Wells–Dawson polyoxoanion [P_2_W_18_O_62_]^6–^ (abbreviated as **{P**_**2**_**W**_**18**_**}**) can be reversibly reduced by 18 electrons per anion in aqueous solution.^17^ The proton-coupled redox activity of Li_6_[P_2_W_18_O_62_] (**Li-{P**_**2**_**W**_**18**_**}**) was exploited in a proof-of-concept paper constructing polyoxometalate-based redox-flow batteries with energy densities of 225 W h L^–1^, allowing for the rapid on-demand generation of hydrogen from water as part of a decoupled electrolysis system. Hence, in the acidic aqueous solutions of the **Li-{P**_**2**_**W**_**18**_**}** salt with concentrations close to the solubility limit (100 mM), the polyoxoanion can experience a series of multi-electron redox processes to yield the super-reduced protonated species **H**_**n**_**{P**_**2**_**W**_**18**_**}-18e (**H_n_[P_2_W_18_O_62_]^(24-*n*)−^ where all 18W^VI^ are reduced by one electron to 18W^V^ in a range potential gap of 800 mV, which is significantly lower than that reported in previous studies under different chemical conditions.^18^

The initial reduction steps of a fully oxidized Li_6_[P_2_W_18_O_62_] (**Li-{P**_**2**_**W**_**18**_**}**) solution at low concentrations were analyzed at pH 7 and 4. In the neutral solution of **Li-{P**_**2**_**W**_**18**_**}**, four one-electron reversible waves were observed in the range of +0.6 and −0.6 V, whereas two one-electron followed by a couple of two-electron waves could be appreciated within the same potential window at pH 4.^17^

In the following article, we present for the first time a study to disentangle the secret of electron stabilization in super-reduced POM clusters. Initially, we present the molecular orbitals accessible in reduced states for **{P**_**2**_**W**_**18**_**}**, together with the galvanostatic discharge curves for Li, Na, and K salts of **{P**_**2**_**W**_**18**_**}**. To fully understand the electronic structure and collective behavior of the reduced species, we have relied on small-angle X-ray scattering (SAXS), density functional theory (DFT), and molecular dynamics (MD) simulations. Collectively, these techniques point out to aggregation and protonation of the clusters as two complementary phenomena that stabilize their negative charge. To further determine the physical characteristics of the super-reduced lithium salts of **{P**_**2**_**W**_**18**_**},** we performed magnetic susceptibility and spectroscopic analyses, including UV–vis, SQUID, and electron paramagnetic resonance measurements. Finally, we present the generalization of the super-reduction process in K salts of anions [P_5_W_30_O_110_]^15–^ and [P_8_W_48_O_184_]^40–^.

## Results and Discussion

### Mechanism of Reduction

**Figure 1:**
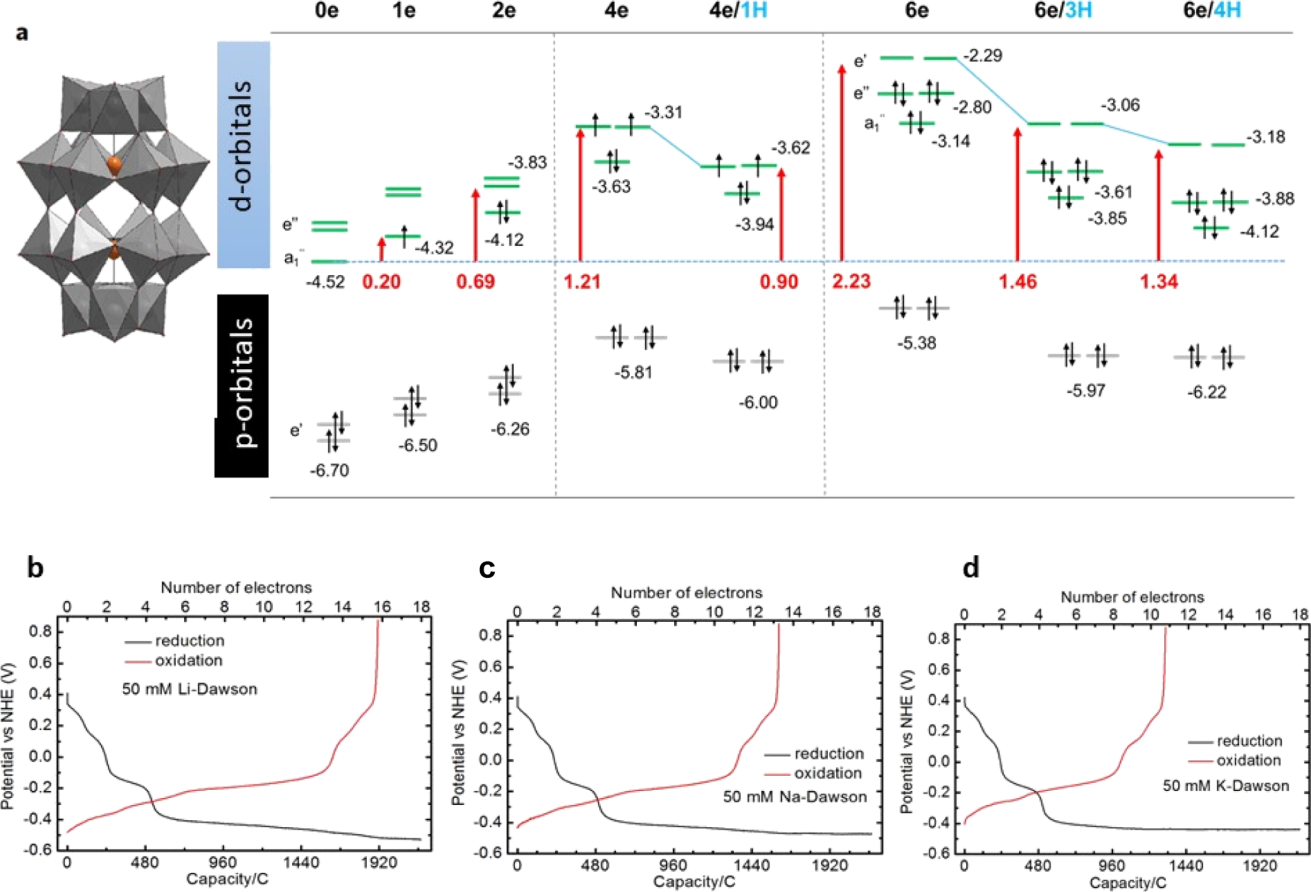
Super-reduced polyoxometalates blueprint data. (a) Structure and frontier molecular orbital (MO) energies for different reduction and protonation states of [P_2_W_18_O_62_]^6–^ (abbreviated as {**P**_**2**_**W**_**18**_}) cluster. Level energies in red and green represent oxo and *d*(W) orbitals, respectively, see text. All energy values (eV) represented in the diagram were computed with the BP86 functional and a Slater TZP basis set (further details in the Computational section, Supporting Information). (b) Galvanostatic discharge curves for the reduction and reoxidation of a 50 mM Li_6_[P_2_W_18_O_62_] solution with a constant current density of ±50 mA cm^–2^ (±648 mA), showing 16 equiv of electrons per cluster. (c) Same experiment with 50 mM Na_6_[P_2_W_18_O_62_] solution with a constant current density of ±50 mA cm^–2^ (±648 mA) showing 13 equiv of electrons per cluster. (d) Same experiment with 50 mM K_6_[P_2_W_18_O_62_] solution with a constant current density of ±50 mA cm^–2^ (±648 mA). Showing 11 equivalents of electrons per cluster.

The electronic structure of POMs has been extensively studied over the last decades by means of computational methods.^19^ In a classical structure such as [P_2_W_18_O_62_]^6–^, tungsten atoms are found in a distorted octahedral environment that makes *d*_*xy*_ orbitals the lowest in energy, followed by degenerated sets of *d*_*xz*_ and d_*yz*_ orbitals. As shown in Figure 1a, the first and second additional electrons are accommodated in a MO of a_1_″ symmetry, whereas the third and fourth are incorporated in two degenerated e″ orbitals. We also provide here the coulombic efficiency in X_6_[P_2_W_18_O_62_] being X = Li, Na, and K. Our results show that the super-reduction of the POM remains independent of the countercation up to 10e per cluster (Figure 1b–d), although the capacity of the POM to store electrons is strongly influenced by the size of the countercations increasing in the order K^+^ < Na^+^ < Li^+^.

Reproducing absolute reduction potentials of POMs is still quite inaccessible to computational methods. Nevertheless, relative values between successive reductions are better estimated (Table S8-1). For POMs, a suitable qualitative analysis can be usually performed from the energy of the MOs to be populated. Figure 1a shows the frontier MOs for different reduction states of {P_2_W_18_}. As expected, MOs shift to higher energies with each electron addition. For instance, the energy of the LUMO shifts from −4.52 eV in the fully oxidized anion to −2.22 eV after reducing it with six electrons. Such destabilization is significantly less important if the electron addition is coupled with the protonation of the POM. When comparing the experimental and computed redox potentials, we cannot unequivocally distinguish the number of protons for each reduction state, but {P_2_W_18_}-6e species should have at least three or four protons attached to the POM framework since otherwise the reduction potentials would become excessively negative (see SI for further details). Note that the LUMO of H_4_{P_2_W_18_}-6e, namely H_4_[P_2_W_18_O_62_]^8–^, would be only +1.34 eV above the LUMO of the fully oxidized {P_2_W_18_} species. This moderate increase in combination with the effect induced by POM aggregation (vide infra) ensures the ability of the Wells–Dawson anion to be reduced multiple times.

**Figure 2:**
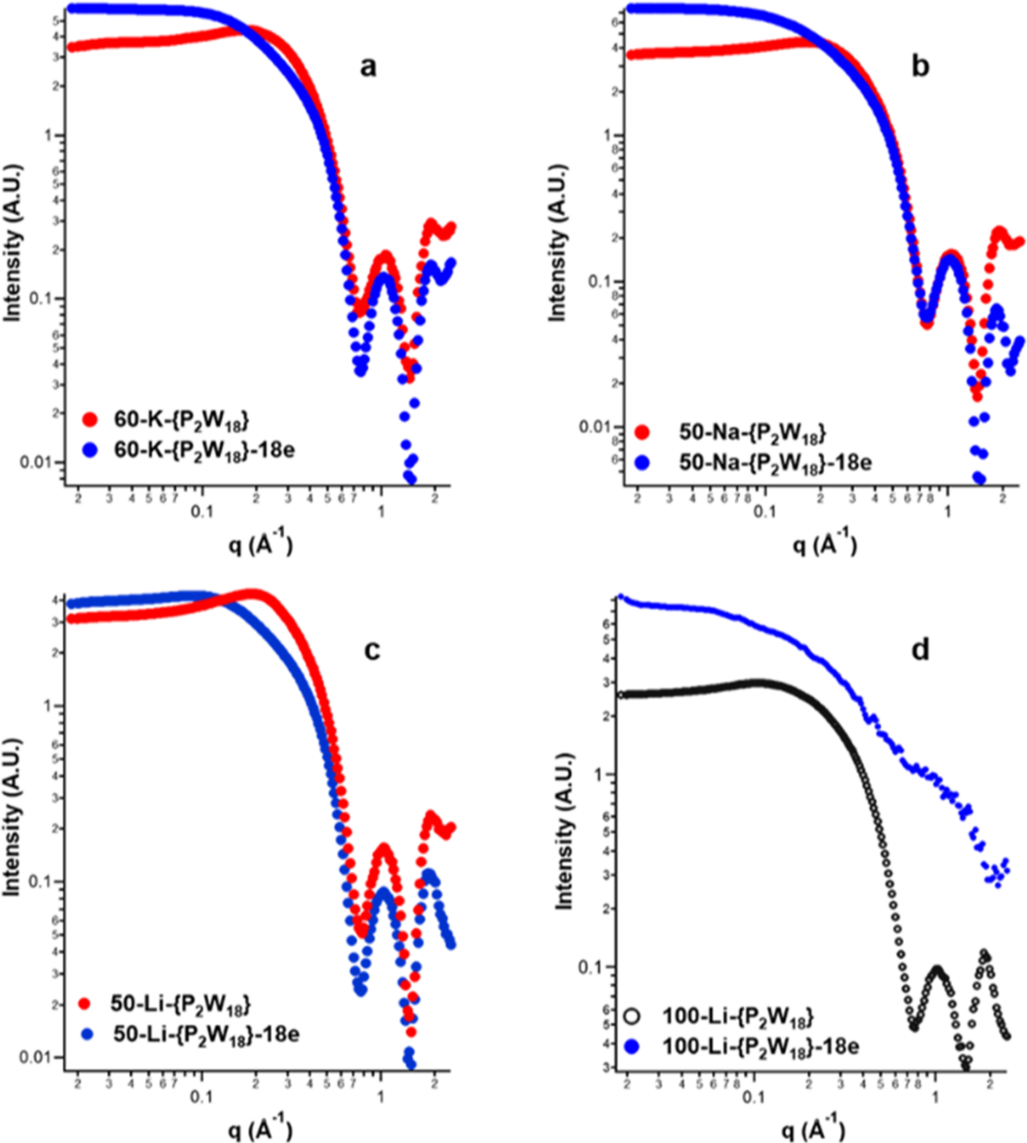
SAXS spectra of {P_2_W_18_} solutions exhibiting differences in the supramolecular assembly. (a) K-{P_2_W_18_} fully oxidized and fully reduced; (b) Na-{P_2_W_18_} fully oxidized and fully reduced; and (c) Li-{P_2_W_18_} fully oxidized and fully reduced. Solutions shown in (a–c) are similar in concentrations for direct comparison; (d) Li-{P_2_W_18_} fully oxidized and fully reduced at 100 mMolar, demonstrating formation of large aggregates upon reduction.

The SAXS spectra for all the studied {P_2_W_18_} solutions are compiled in Figure 2 and solution descriptions are in Table 1. The Li-series is more extensively studied with intermediate reduction states between fully oxidized {P_2_W_18_} and fully reduced {P_2_W_18_}-18e because this series displays an unusual scattering phenomenon. The Li and K solutions were diluted from the 100 mMolar solutions, and the 60 mMolar K solution is close to its maximum solubility. Nonetheless, these concentrations can be compared directly for this discussion. Notably, for all the countercations, there is a distinct coulombic peak (between *q* ∼ 0.1 and 0.3 Å^–1^) for the fully oxidized [P_2_W_18_O_62_]^6–^, which is eliminated for 50-Na-{P_2_W_18_}-18e and 60-K-{P_2_W_18_}-18e, and partially eliminated for 50-Li-{P_2_W_18_}-18e. This coulombic peak indicates ordering in solution created by repulsion between the polyanions where the repulsion is inadequately shielded by the countercations that are present only in stoichiometric quantities. The peak can generally be eliminated with addition of excess electrolytes.^31,32^ All solutions contain only six equivalents of the alkali per cluster; the electrochemical reduction is performed in 1 M H_2_SO_4_ solutions. The scattering curves for the fully oxidized solutions (Figure 2a–c) were fitted with three parameters describing the size of the clusters and degree of ordering (Table 1 and Figure 2a–c). These parameters are nearly identical for the three solutions and suggest that Li^+^, Na^+^, and K^+^ similarly exhibit minimal interactions with [P_2_W_18_O_62_]^6–^ in these solutions. The radius of 5.7 Å is in good agreement with the physical diameters of the slightly oblong cluster shape (∼11 × 14 Å, oxygen to oxygen distances). Also, it is notable that, in these solutions (and most of the prepared solutions), two oscillations (*q* > 0.7 Å^–1^, Figure 2) are observed that agree well with the simulated scattering data of [P_2_W_18_O_62_]^6–^. This indicates the solutions are pure and monospecific, containing only [P_2_W_18_O_62_]^6–^ POMs.

**Table 1:** Fitting Form and Structure Factors for SAXS of Fully Oxidized {P_2_W_18_} Solutions^b^

formula	conc. (mMolar)	cluster radius (Å)	Eta^a^ (Å)	Phi^b^
Li_6_[P_2_W_18_O_62_]	50	5.7	24	0.7
50-Li-{P_2_W_18_}				
Na_6_[P_2_W_18_O_62_]	50	5.7	23	0.5
50-Na-{P_2_W_18_}				
K_6_[P_2_W_18_O_62_]	60	5.7	22	0.6
60-K-{P_2_W_18_}				

a Half of the center-to-center distance of clusters. A unit-less term that describes the “pack” of the clusters, or how many nearest neighbors surround each cluster (larger number indicates more “nearest neighbor” clusters).

b See Figures S4-SAXS, S5-SAXS, and S6-SAXS for the data fits.

It seems counterintuitive that the anion–anion repulsion is greatly diminished upon 16-electron reduction. This suggests that the alkali-countercations become more closely associated with reduction, partially neutralizing and shielding the negative charge. The aforementioned coulombic peak between *q* ∼ 0.1–0.3 Å^–1^ is not completely eliminated in the 50-Li-{P_2_W_18_}-18e solution. The degree of elimination of the coulombic peak can be evaluated by comparing the Δ*I*_0_ (at minimal *q*) between the scattering curves for X-A-{P_2_W_18_} and X-Li-{P_2_W_18_}-18e. ΔI_0_ is approximately 4 A.U. (arbitrary units) for A = Na and K, and <1 for A = Li. Li^+^ is much smaller than Na/K^+^, meaning it carries a larger hydration sphere. Therefore, Li^+^ undergoes considerably less direct contact ion-pairing than Na/K^+^, diminishing its ability to partially neutralize the high negative charge of [P_2_W_18_O_62_]^24–^ ({P_2_W_18_}-18e).

The strong (high intensity) and featureless scattering curve for 100-Li-{P_2_W_18_}-18e resembles surface scattering of an amorphous solid, Figure 2d, yet the solution remains completely dissolved, with no evidence of precipitation. The 100-Li-{P_2_W_18_}-6e and 100-Li-{P_2_W_18_}-3e solutions exhibit similar phenomena but to a progressively lesser extent (Figure S6-1-SAX). Cu-K_γ_ X-rays cannot sufficiently interact with the 100-Li-{P_2_W_18_}-18e solution to observe the scattering species. However, they can sufficiently interact with the {P_2_W_18_} clusters of the 100-Li-{P_2_W_18_}-0e solution; the characteristic features of the clusters are observable (Figure 2c). The only difference between these solutions is the 18 valence electrons per cluster, which scatter weakly compared to the 1242 core electrons of the 18 W-ions per cluster.^20,21^

**Figure 3:**
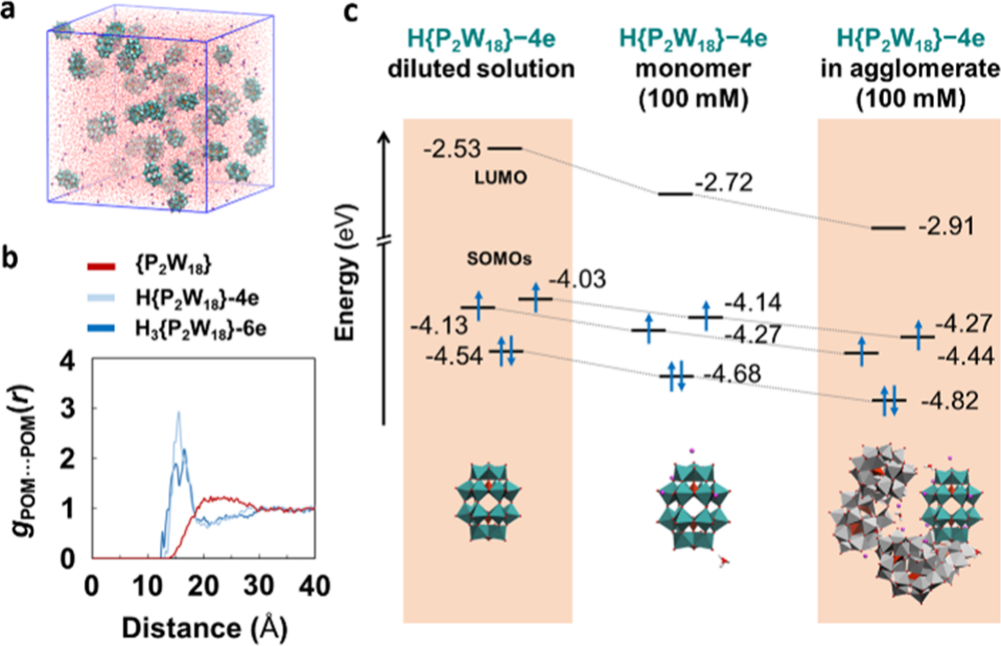
Electronic properties and collective behavior of Wells–Dawson anions at initial reduction states. (a) Snapshot of a representative 3D-periodic simulation box used for classical MD simulations (see the Computational Details section for further details). (b) POM···POM radial distribution functions (RDFs) computed from classical MD simulations taking as reference the center of mass of each POM. Red, light blue, and dark blue lines denote simulations with [{P_2_W_18_}]^6–^, [H{P_2_W_18_}-4e]^9–^_,_ and [H_3_{P_2_W_18_}-6e]^9–^ anions, respectively. RDFs were averaged over the last 10 ns of 40 ns simulations and sampling data every 2 ps. (c) Schematic MO diagram showing the stabilizing effect of agglomeration on the MOs of H{P_2_W_18_}-4e. Energies (in eV) were computed for the POM highlighted in cyan in the snapshots using the hybrid-GGA B3LYP functional and a DZP-quality basis set. Solvent effects (water) were included through the IEF-PCM model.

To better understand the collective behavior of Li_6_[P_2_W_18_O_62_] salt and how it relates with the super-reduction process, we conducted MD simulations for several reduction states of {P_2_W_18_} in aqueous solutions (Figure 3a). Initially, the behavior of the partially reduced [H{P_2_W_18_}-4e]^9–^, [H_2_{P_2_W_18_}-4e]^8–^_,_ and [H_3_{P_2_W_18_}-6e]^9–^ anions was compared to that of fully oxidized {P_2_W_18_} anions at ca. 100 mM and in the presence of hydronium cations to mimic the experimental conditions. In line with the SAXS measurements, the {P_2_W_18_}···{P_2_W_18_} RDF (Figure 3b, red curve) does not show any peak, indicating a complete lack of agglomeration. However, those for reduced POMs display an array of peaks between 12.6 and 18.9 Å, revealing a range of preferred intermolecular distances between reduced anions in which POMs mainly interact via lithium- and hydronium-mediated contacts caused by the increased negative charge of POM clusters that promote the formation of ion pairs. Direct hydrogen bonds between anions and water-mediated contacts were also reported. Taking the case of H{P_2_W_18_}-4e as a representative example, we evaluated the influence of the observed agglomeration on the electronic structure of the POMs. Interestingly, when H{P_2_W_18_}-4e participates in the supramolecular assembly, its LUMO is stabilized by a non-negligible ∼380 mV. This phenomenon facilitates the injection of electrons in the system at lower potentials, which can explain the unconventional redox properties of concentrated {P_2_W_18_} solutions. The stabilizing effect induced by agglomeration was ascribed to a remarkable increase in the effective ion-pairing. The incorporation of explicit cations besides the dielectric continuous solvent model already stabilizes the unoccupied MOs by ∼200 mV, and including the POM into a small agglomerate results in an additional stabilization of ∼180 mV (Figure 3c). This observation is in line with the experimental decrease of ∼100 mV in the H{P_2_W_18_}-4e reduction potential when going from 2 to 100 mM solutions.^17^

Overall, these results collectively indicate that both the protonation and agglomeration of partially reduced POMs play a crucial role in the high-reduction process. As the size of the cation decreases, so does the degree of POM···cation pairing due to the stronger hydrophilicity of the cation, as suggested by SAXS spectra (vide supra). Thus, it is reasonable to think that less intense ion-pairing triggers the association of a higher number of protons to POM clusters to compensate for the negative charge that increases with each reduction step. Since the impact of protonation on the MO stability is much more important than that of non-covalent ion-pairing, a moderate rather than strong ion-pairing is expected to facilitate further reduction steps, explaining why the capacity of {P_2_W_18_} is maximized with Li^+^ salts.

### Characterization of the Super-Reduced Species

**Figure 4:**
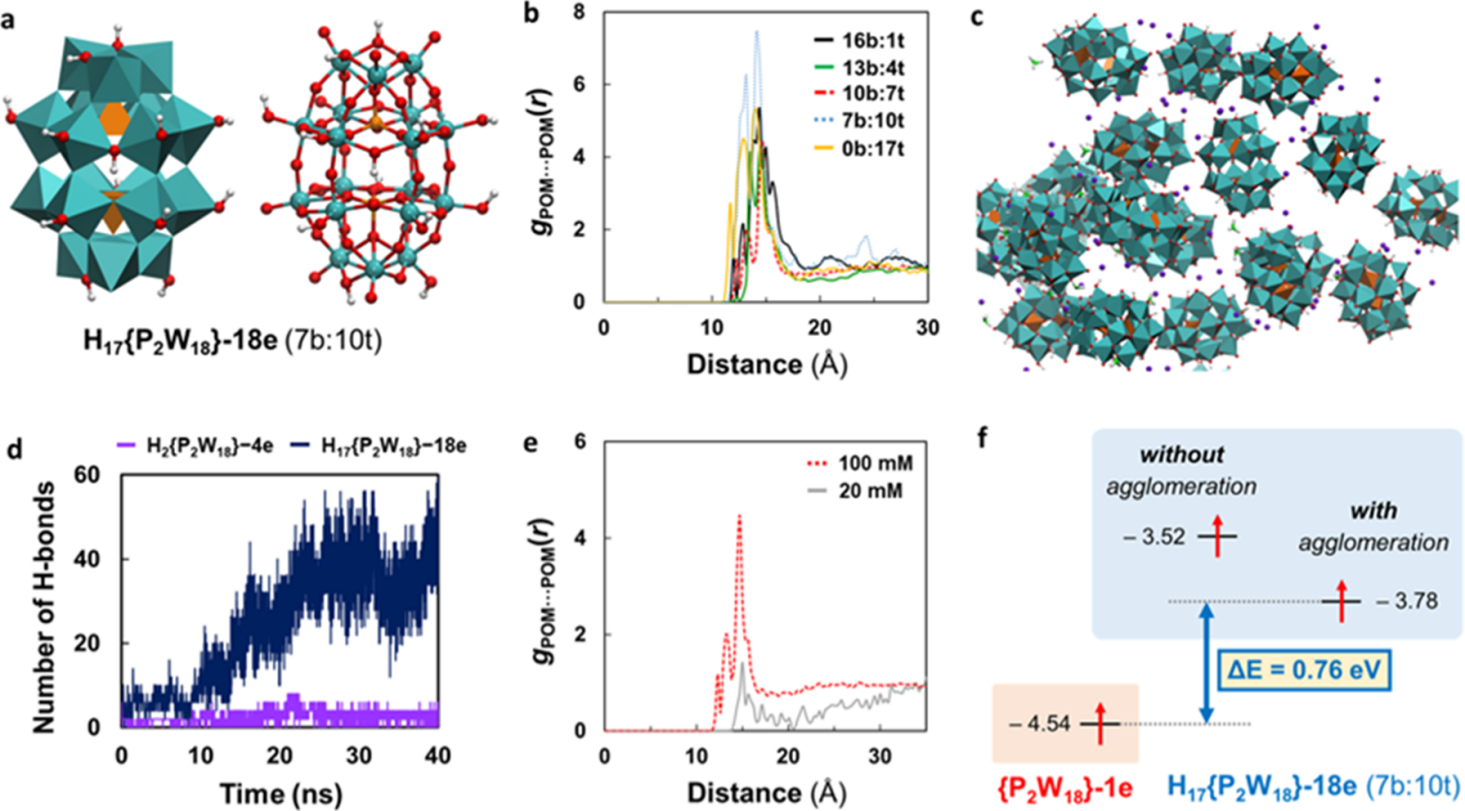
Electronic properties and collective behavior for the super-reduced [P_2_W_18_O_62_]^24^^–^ anion. (a) Polyhedral and balls-and-sticks representation of anion H_17_{P_2_W_18_}-18e (7b:10t), bearing seven and ten protons at bridging and terminal oxygen atoms. This proton distribution was found to be the most likely distribution for a system with 17 protons, although other distributions can coexist under the experimental conditions (Table S8-2). (b) Comparison of the POM···POM RDF for several H_17_{P_2_W_18_}-18e anions with different bridging/terminal ratios (Table S8-2), obtained from MD simulations of 100 mM POM solutions. (c) Snapshot of a H_17_{P_2_W_18_}-18e (7b:10t) agglomerate at the last step of the simulation. POMs are represented as polyhedra, Li cations as purple spheres, and hydronium cations as sticks with O atoms highlighted in green. Water molecules are omitted for clarity. (d) Evolution of the number of hydrogen bonds between POMs computed over 40 ns of simulation for H_17_{P_2_W_18_}-18e (7b:10t) (blue line) and H_2_{P_2_W_18_}-4e (purple line), highlighting that direct H-bonding arises as a non-negligible cohesion agent in super-reduced anions. (e) Comparison of the POM···POM RDF at different concentrations using the H_17_{P_2_W_18_}-18e (10b:7t) anion as a representative example. The simulation at a high concentration revealed an average number of 1.34 POMs in close contact with another POM, whereas at low concentrations, the average number of neighbors drops to 0.06 POMs, in agreement with the experimental concentration dependence. (f) Schematic MO diagram comparing the energy levels of the SOMO of {P_2_W_18_}-1e with the highest SOMO of H_17_{P_2_W_18_}-18e (7b:10t) in solution (non-associated monomer) and within an agglomerate structure (Figure S8-9).

Additional calculations were carried out to propose a plausible structure for the super-reduced anion (Figure 4). DFT-MD simulations of a H_18_{P_2_W_18_}-18e cluster in solution revealed the spontaneous migration of one proton from a bridging to a terminal oxygen, as well as an overall protonation degree oscillating between 16 and 17 protons during the 6.5 ps trajectory (Figure S8-2). Using a representative H_17_{P_2_W_18_}-18e structure obtained from the DFT-MD trajectory, iterative optimization of the structure and the wave-function in different spin states locate one electron on each W center, combining a population of d_*xy*_-like orbitals with d_*xz*_/d_*yz*_ ones for protonations at terminal sites. These metal electrons were predicted to be unpaired but magnetically coupled to some extent, with an open-shell singlet being the most likely configuration, followed by quintet and triplet states, lying at only +1.5 and +1.6 kcal·mol^–1^_,_ respectively (Figures S8-6).

Further exploration was aimed at evaluating the proneness of H_17_{P_2_W_18_}-18e to bear protons at terminal positions. These revealed that, indeed, the super-reduced cluster might combine protons at bridging and terminal oxygen sites. Specifically, the most likely proton distribution should be close to 7 protons at bridging positions plus 10 at terminal ones (7b:10t) (Figure 4a and Table S8-2), although other proton distributions might be accessible when POMs are not isolated monomers but a part of supramolecular assemblies. Even so, large agglomerates were similarly observed for any proton distribution (Figure 4b–e), which might explain the unusual SAXS scattering recorded for this species (Figure 2). Most importantly, the energy of the highest SOMO of H_17_{P_2_W_18_}-18e (7b:10t) in the agglomerate is only 0.76 eV higher than the SOMO of the 1e-reduced {P_2_W_18_}-1e (−4.54 eV) computed at the same level of theory (Figure 4f), which fully agrees with the observed voltage window of 0.8 V for the reoxidation process.

**Figure 5:**
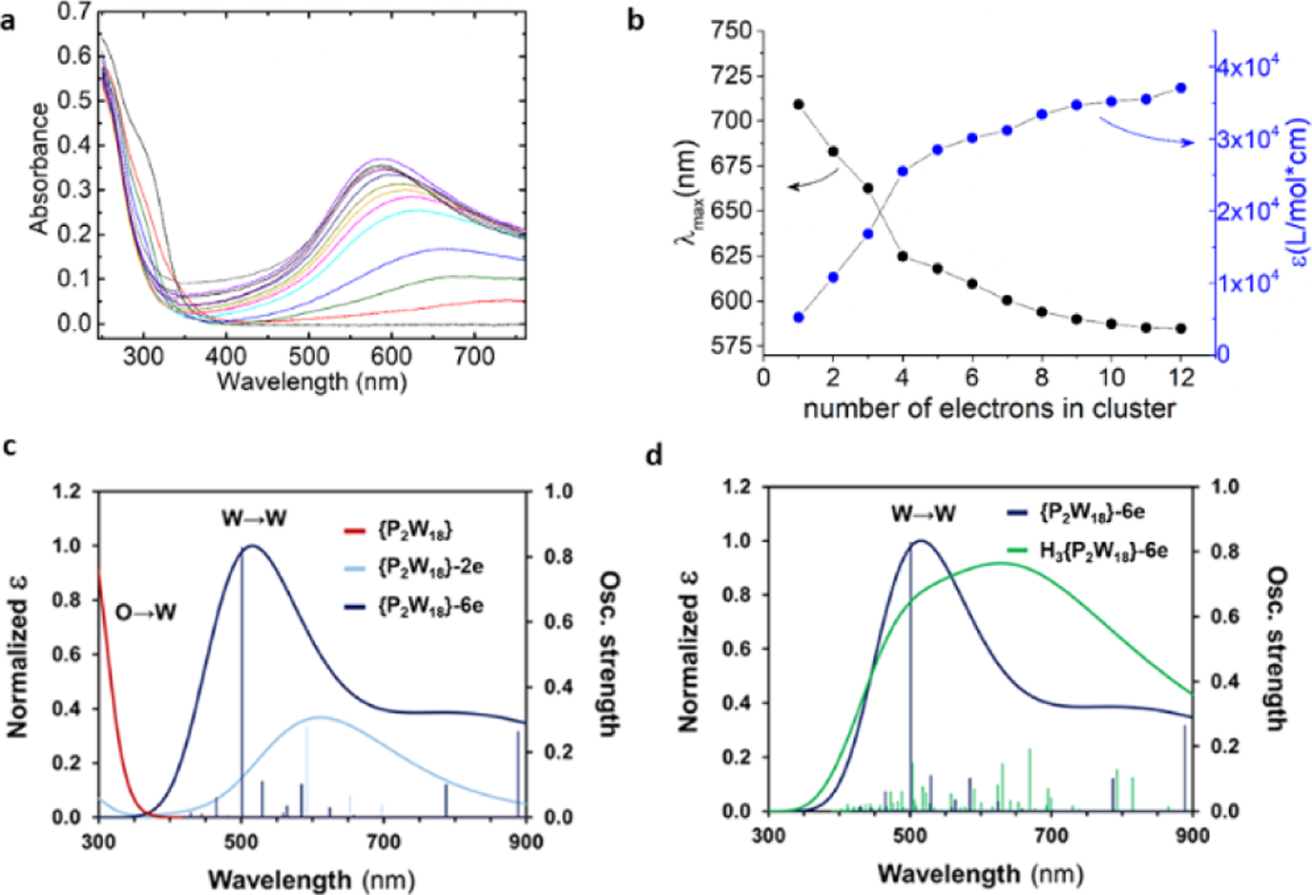
Comparative data sets for Ultraviolet–visible (UV–vis) experimental and computational that describes the Li-{P_2_W_18_} cluster. Redox flow electrolysis cell results from 0–12 electrons in cluster for a 10 mM of Li-{P_2_W_18_}, namely Li_6_[P_2_W_18_O_62_], in water, (a,b) UV–vis data, each line represents an increase in voltage applied to the bias equivalent to the reduction of Li-{P_2_W_18_}, see Supporting Information. (c) Computed UV–vis spectra for the fully oxidized {P_2_W_18_} anion (red line) and the 2 and 6 electron-reduced forms (light and dark blue, respectively) and (d) Effect of protonation in the UV–vis spectrum of {P_2_W_18_}-6e.

In line with the conclusions inferred from theoretical data, experimental absorption spectra support the partial occupation of the *d*(W) orbitals, which causes the appearance of a deep blue coloration in the solution and the corresponding band in the UV–vis spectrum at ∼650 nm associated with transitions of *d*(W) → *d*(W) (Figure 5). We have been able to experimentally follow the reductions of Li-{P_2_W_18_} in a purpose-built e-chem UV–vis cell (see Supporting Information for details) and plot the absorbance (λ) as a function of the number of electrons per cluster (Figure 5b). This is a unique result where the electron-storage capacity of a material, normally limited to 1–2 electrons per molecule, is not only increased to 12 electrons per molecule but can also can be measured by a physical property such as an increase in UV–vis. This property of Li-{P_2_W_18_} shows great promise as it can perform reversible multi-electron reactions with high structural stability in aqueous media; each subsequent reduction was followed by high precision UV–vis measurements.

To explain the evolution of the experimental UV–vis spectrum of Li-{P_2_W_18_} upon reduction, we simulated the absorption spectra of the cluster with 0, 2, and 6 extra electrons (Figure 5c). As in the experimental spectrum, we observed that the band at *ca.* 300 nm in the spectrum of the fully oxidized species associated with *p*(O) → *d*(W) transitions decreases its intensity in the spectrum of the 2e-reduced one and completely disappears after further reducing the system. This is caused by the effect of populating the lowest *d*(W) orbitals, preventing the transitions from the oxo band to these orbitals. Also, in agreement with the experimental data, the simulated spectrum of {P_2_W_18_}-2e reveals a band centered at *ca.* 600 nm associated with *d*(W) → *d*(W) transitions, which is shifted to more energetic transitions with subsequent reductions. See Supporting Information for further details.

**Figure 6:**
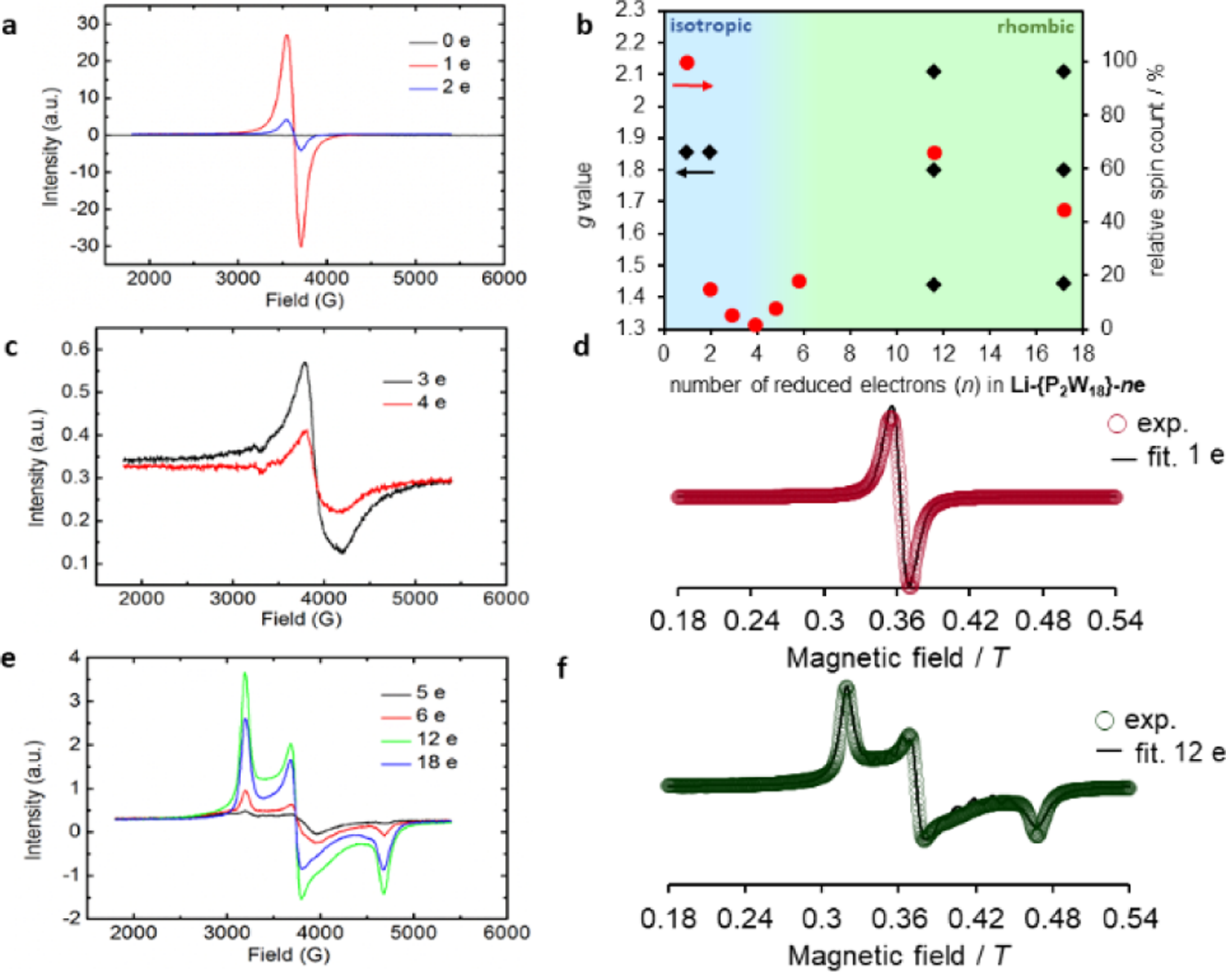
EPR results of 100 mM Li-{P_2_W_18_} salt at different reduction states at *T* = 100 K. (a) EPR for the Li-{P_2_W_18_} sample, corresponding to the applied current for 1 and 2 electron-reduced samples. (b) Different g values for multiple reduced Li-{P_2_W_18_} samples. Different *g* values corresponding to two types of W atom environments in the cluster. From species reduced between 1-5e, electron density is located around 12 W in the belt region; beyond that (6–18e), the electron density is also distributed around the 6 W cap, and clusters are protonated and aggregated. (c) Signal corresponding to 3 and 4 e– reduced samples. (d) Theoretical EPR fitting for 1 e– reduced Li-{P_2_W_18_} spectra. Finally, (e) signals for 5–18 electron-reduced Li-{P_2_W_18_} samples. (f) Theoretical EPR fitting for 12 electron-reduced Li-{P_2_W_18_} EPR spectra.

To reveal the magnetic properties of reduced Li-{P_2_W_18_}, the EPR spectra of frozen solutions of Li-{P_2_W_18_}-*n*e (100 mM; *n* = 1, 2, 3, 4, 5, 6, 12, and 17) were measured. Interestingly, EPR signals of Li-{P_2_W_18_}-*n*e drastically changed from isotropic (*n* = 1–4) to rhombic (*n* = 5–17) (Figure 6). The EPR spectrum of Li-{P_2_W_18_}-1e showed the isotropic signal at *g* = 1.856, which was in good agreement with the reported *g* value (1.852) of a 1e-reduced Wells–Dawson-type POM.^22^ The slightly small *g* value of Li-{P_2_W_18_}-1e was consistent with the observation of slightly small direct current magnetic susceptibility of Li-{P_2_W_18_}-1e (1.6 μ_B_) due to the strong spin–orbit coupling of W^5+^ (see in Supporting Information-5), also supporting the presence of W^5+^ species observed by UV–vis spectra (Figure 5). The signal intensities decreased with increasing the number of reduced electrons (*n* = 1–4), which was presumably interpreted by super-exchange interactions between W^5+^ species to form coupled EPR silent species^23^ and disproportionation reactions between, for example, 2Li-{P_2_W_18_}-3e and Li-{P_2_W_18_}-2e + Li-{P_2_W_18_}-4e via an outer sphere electron transfer in concentrated solutions of Li-{P_2_W_18_}. In fact, DFT calculations show that this process can be energetically accessible with a Δ*G*° of +1.3 kcal·mol^–1^.

On the other hand, the EPR spectrum of Li-{P_2_W_18_}-17e could be fitted by the following *g* factors; *g*_*x*_ = 2.108, *g*_*y*_ = 1.800, and *g*_*z*_ = 1.442, illustrating the rhombic signal (Figure 6). The unusual rhombic EPR signals of Li-{P_2_W_18_}-*n*e (*n* = 5–17) would be explained by the unique protonation behavior of highly reduced Li-{P_2_W_18_}, which was found to accommodate some protons at terminal W=O sites (Figure 4a). The formation of distorted octahedral W^5+^–OH species resulted in the elongation of the W–O bond and the modification of the orbital occupation from *d*_xy_ in non-protonated O terminal sites to *d*_xz_/*d*_yz_ in protonated sites. The signal intensity increased with increasing the number of reduced electrons (*n* = 5–12) presumably because of the increase in W^5+^–OH units. However, further studies would be required to understand the decrease of the signal intensity observed from *n* = 12–18, likely due to the coupling between W^5+^ ions.^24,25^ The rhombic EPR signals together with the increase (*n* = 5–12) and decrease (*n* = 12–17) in signal intensities could also be explained by the formation of W–W bonds, as reported for other systems under different chemical conditions.^5,26^ However, for highly reduced and protonated Wells–Dawson anions, DFT calculations suggest that the formation of W–W bonds is thermodynamically unfavorable (see Supporting Information-8). In fact, the putative formation of metal–metal bonds would permit the reduction of Wells–Dawson clusters beyond 18 electrons, as reported for Keggin anions.^5,27,28^ Indeed, a very recent report^29^ showed that the formation of Mo–Mo bonds in the PMo_12_ framework only occurs after the full 1e-reduction of all the Mo centers, locating the extra electrons in metallic bonds. The possible role of the metal–metal bond in the irreversible reduction of POMs was already pointed out by Launay in 1976.^30^ The results presented strongly suggest that the highly reduced Li-{P_2_W_18_}-*n*e (*n* = 5–18) possesses meta-stable W^5+^ species under a high concentration condition that can readily react with protons to generate hydrogen gas when the solution is diluted.^17^

**Figure 7:**
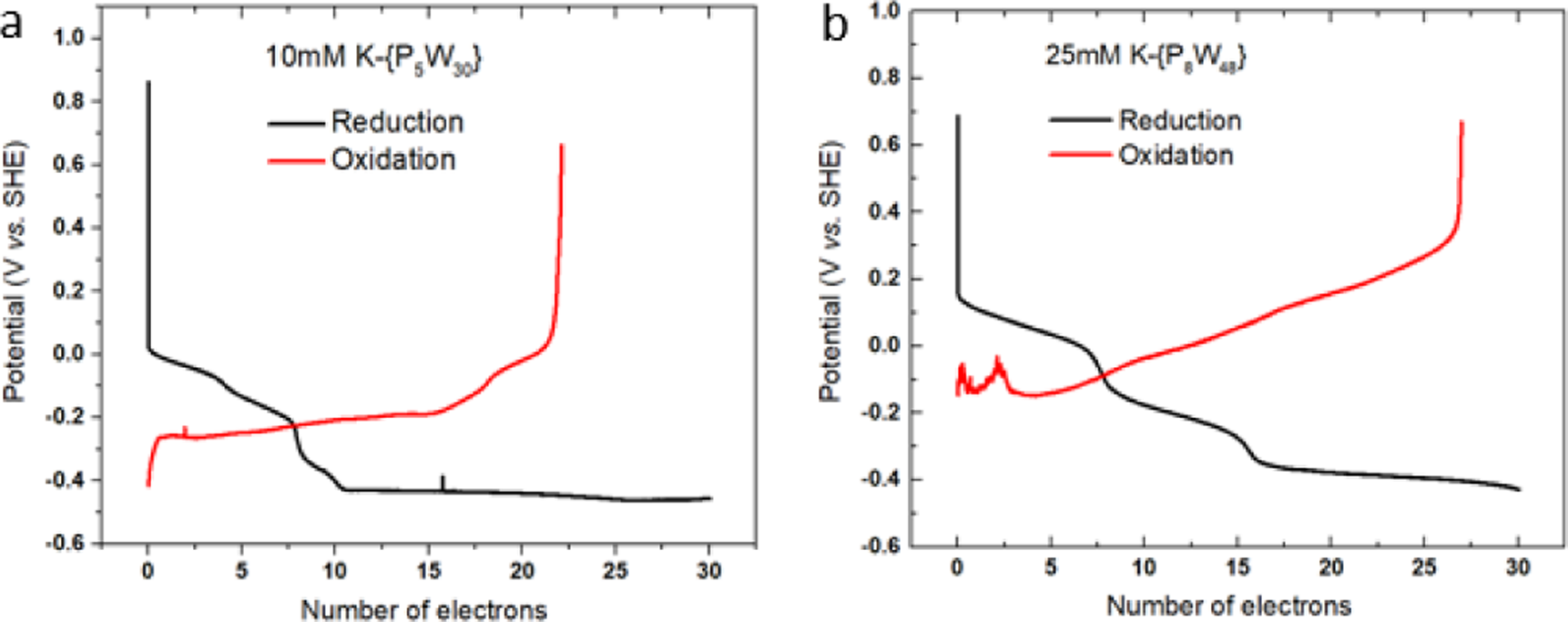
Galvanostatic discharge curves for the reduction and reoxidation of K salts of {P_5_W_30_} and -{P_8_W_48_} anions. (a) 23 e–reduction/reoxidation curves of a 10 mM solution of K-{P_5_W_30_} and (b) 27 e–reduction/reoxidation curves of a 25 mM solution of K-{P_8_W_48_} and battery testing devices were heated to 70 °C to maintain the solubility.

Finally, we investigated the electron-storage ability of larger [P_5_W_30_O_110_]^15–^ ({P_5_W_30_}) and [P_8_W_48_O_184_]^40–^ ({P_8_W_48_}) anions, see Figure 7. Solubility was a significant challenge here, as POM solubility generally decreases as the anion and charge/metal ratio increases from 0.3 for {P_2_W_18_}^6–^ to 0.5 for {P_5_W_30_}^15–^ and 0.83 for {P_8_W_48_}^40–^. This meant the high concentrations (*ca* 100 mM) shown to yield the novel behavior for Li-{P_2_W_18_} could not be achieved. However, the aggregation still works within the solubility limit for these two clusters. Our preliminary results show that upon charging a 10 mM solution of K-{P_5_W_30_} by 30 electrons per cluster, 23 electrons could be released, representing 77% coulombic efficiency compared with 58% at the same concentration for {P_2_W_18_}. For K-{P_8_W_48_} solubilities of >10 mM could only be achieved at elevated temperatures, but at 70 °C, charging a 25 mM solution of K-{P_8_W_48_} by 30 electrons per cluster allowed for the storage of 27 electrons.

## Conclusions

The intricate mechanism responsible for the super-reduction of fully inorganic polyoxometalate salts with concentrations close to the solubility limit was investigated using a variety of experimental and computational techniques and the recently reported case of {P_2_W_18_}. Analyses of the electronic structure and collective behavior in aqueous solution along the charging process revealed that the protonation of the POMs and their agglomeration in solution via cation-mediated contacts are complementary factors to promote the formation of super-reduced species. Both phenomena induce the stabilization of the empty d(W) orbitals allowing the incorporation of many electrons at low potentials. As such, this process is highly countercation-dependent since the size and, in turn, the hydrophilicity of the countercation can modulate the energy levels of the POM via balancing the magnitude of protonation and ion-pairing effects, explaining the greater reduction capacity of lithium salts compared to sodium or potassium ones. The complexity of the EPR spectra would suggest that these materials may undergo disproportion at a certain reduction state. The electronic structure and the relative high robustness of the protonated {P_2_W_18_}, {P_5_W_30_}, and {P_8_W_48_} frameworks very probably prevent the formation of metal–metal bonds and limit the reduction to one electron per metal center, which in turn allows reversible oxi-reduction processes of only 800 mV in the case of {P_2_W_18_}.^17^ This work represents the first attempt to understand the mechanism of super-reduction of polyoxometalates in specific acidic conditions. More efforts are underway in our laboratories to further characterize the super-reduced species.

## Supporting Information Available

Material synthesis and characterization; comparison with different countercations of the W–Dawson cluster; electron paramagnetic resonance; paramagnetic 183w NMR spectroscopy (183w NMR) of Li Dawson at different reduced states; superconducting quantum interference device (squid) data; small-angle X-ray scattering (saxs) data; UV-vis device; theoretical analysis of the super-reduced Wells–Dawson polyoxotungstate; and computational details (PDF)

## Abbreviations

{P_2_W_18_}: The Wells–Dawson phosphotungstate anion with the formula [P_2_W_18_O_62_]^6–^
X_6_[P_2_W_18_O_62_]: X = Li, Na, and K as Li-{P_2_W_18_}, Na-{P_2_W_18_}, and K-{P_2_W_18_}
[P_2_W_18_O_62_]^24–^: {P_2_W_18_}-18e is the fully reduced species
H_n_{P_2_W_18_}-18e: H_n_[P_2_W_18_O_62_]^(24-n)−^ as
50-Li-{P_2_W_18_}: 50 mM solution of Li_6_[P_2_W_18_O_62_]
50-Na-{P_2_W_18_}: 50 mM solution of Na_6_[P_2_W_18_O_62_]
60-K-{P_2_W_18_}: 60 mM solution of K_6_[P_2_W_18_O_62_]
H_4_{P_2_W_18_}-6e: H_4_[P_2_W_18_O_62_]^8–^
100-Li-{P_2_W_18_}-18e: 100 mM solution of fully reduced Li_6_[P_2_W_18_O_62_]
100-Li-{P_2_W_18_}-6e: 100 mM solution of six electron reduced Li_6_[P_2_W_18_O_62_] salt
100-Li-{P_2_W_18_}-3e: 100 mM solution of three electron reduced Li_6_[P_2_W_18_O_62_] salt
100-Li-{P_2_W_18_}-0e: 100 mM solution of zero electron reduced Li_6_[P_2_W_18_O_62_] salt
[H{P_2_W_18_}-4e]^9–^: partially reduced species: four electron reduced mono-protonated species H[P_2_W^V^_4_W^VI^_14_O_62_]^9–^
[H_2_{P_2_W_18_}-4e]^8–^: di-protonated four electron reduced H_2_[P_2_W^V^_4_W^VI^_14_O_62_]^8–^
[H_3_{P_2_W_18_}-6e]^9–^: tri-protonated six electron reduced H_3_[P_2_W^V^_6_W^VI^_12_O_62_]^9–^
H_17_{P_2_W_18_}-18e: fully reduced species with 17 protons H_17_[P_2_W_18_O_62_]^7–^
{P_2_W_18_}-1e: one electron reduced [P_2_W^V^W^VI^_17_O_62_]^7–^
H_18_{P_2_W_18_}-18e: fully reduced species with 18 protons H_18_[P_2_W_18_O_62_]^6–^
Li-{P_2_W_18_}-*n*e: number of reduced electrons in a 100 mM solution of Li_6_[P_2_W_18_O_62_] (*n* = 1, 2, 3, 4, 5, 6, 12, and 17), for example, one electron reduced [P_2_W^V^W^VI^_17_O_62_]^7–^ as Li-{P_2_W_18_}-1e, two electron reduced [P_2_W_2_^V^W^VI^_17_O_62_]^8–^ as Li-{P_2_W_18_}-2e, and so forth
{P_5_W_30_}: the Preyssler-type phosphotungstate anion with the formula [P_5_W_30_O_110_]^15–^
{P_8_W_48_}: the wheel-shaped phosphotungstate anion with the formula [P_8_W_48_O_184_]^40–^
K-{P_5_W_30_}: K_15_[P_5_W_30_O_110_]
K-{P_8_W_48_}: K_40_[P_8_W_48_O_184_]
