# Supplementary material for: Effective Storage of Electrons in Water by the Formation of Highly Reduced Polyoxometalate Clusters
Source: J Am Chem Soc. 2022 May 10;144(20):8951–60. doi: 10.1021/jacs.1c10584 (PMC9171825; doi:10.1021/jacs.1c10584)
Supplement: Supplementary file 1 — ja1c10584_si_001.pdf [file ja1c10584_si_001.pdf]

Supporting Information for:

Effective Storage of Electrons in Water by the Formation of Highly Reduced Polyoxometalate Clusters

Jia-Jia Chen,[1]†‡ Laia Vilà-Nadal, [1]‡ Albert Solé-Daura,[2] Greig Chisholm,[1] Takuo Minato, ††[1] Christoph Busche,[1] Tingting Zhao,[1] Balamurugan Kandasamy,[1] Alexey Y. Ganin,[1] Rachelle M. Smith,[3] Ian Colliard,[3] Jorge J. Carbó,[2] Josep M. Poblet, [2]* May Nyman[3]* and Leroy Cronin[1]*

[1]School of Chemistry, the University of Glasgow, University Avenue, Glasgow G12 8QQ, UK.

[2]Department de Química Física i Inorgànica, Universitat Rovira i Virgili, Marcel·lí Domingo 1, 43007 Tarragona, Spain.

[3]Department of Chemistry, Oregon State University, Corvallis, OR 07331-USA

Contents

SI-1: MATERIAL SYNTHESIS AND CHARACTERIZATION 3

SI-2: COMPARISON WITH DIFFERENT COUNTER CATION OF W-DAWSON CLUSTER........................ 6

SI-3: ELECTRON PARAMAGNETIC RESONANCE (EPR) 8

SI-4: PARAMAGNETIC ^{183}W NUCLEAR MAGNETIC RESONANCE SPECTROSCOPY (^{183}W NMR) OF LI-DAWSON AT DIFFERENT REDUCED STATE 10

SI-5: SUPERCONDUCTING QUANTUM INTERFERENCE DEVICE (SQUID) DATA 11

SI-6: SMALL-ANGLE X-RAY SCATTERING (SAXS) DATA 16

SI-7: UV-VIS DEVICE..21

SI-8 THEORETICAL ANALYSIS OF THE SUPER-REDUCED WELLS-DAWSON POLYOXOTUNGSTATE.....24

SI-9 COMPUTATIONAL DETAILS. 40

SI-1: Material synthesis and characterization

Synthesis of α-$Li_6[P_2W_{18}O_{62}]$: The preparation of $Li_6[P_2W_{18}O_{62}]$ was similar to the reported procedure.[1] Crystals obtained from the synthesis were recrystallised from methanol, 1M LiCl solution and finally from water.

Synthesis of $K_6[P_2W_{18}O_{62}]$ and $Na_6[P_2W_{18}O_{62}]$: $K_6[P_2W_{18}O_{62}]$ was prepared via the reported method[2]. And $Na_6[P_2W_{18}O_{62}]$ was prepared by ion-exchange of $K_6[P_2W_{18}O_{62}]$ with $NaClO_4$ since $NaClO_4$ has a much higher solubility than $KClO_4$ water (1.5 g per 100 ml for $KClO_4$ at 25°C while 209.6 g per 100 ml for $NaClO_4$ at 25°C). 240g $K_6[P_2W_{18}O_{62}]$ was dissolved into 200 ml under stirring at 80 °C. 45 g $NaClO_4$ (0.3 mol) was added into the above solution. The precipitate was filtered off and recrystallized in water to produce well-behaved yellow crystals. The element analysis by inductively coupled plasma (ICP-OES 5100, Agilent Technologies) method found: W 62.31%, Na 2.86 %, K 0.26%, and P 1.13 %. The molar ratio of $n_{Na} : n_K$ is around 18.64:1 that means most of the K cations in the W-Dawson clusters are replaced with Na cations.

Synthesis of $K_{14}[NaP_5W_{30}O_{110}]\cdot 22H_2O$: $K_{14}[NaP_5W_{30}O_{110}]\cdot 22H_2O$ was synthesized from a modified procedure.[2] $Na_2WO_4\cdot 2H_2O$ (29.7g) and NaCl (3.51g) were mixed with deionized water in a 125 mL Teflon-lined autoclave, then 21 mL of 85% H_3PO_4 was added as well, the mixture was stirred under room temperature for one hour. The autoclave was placed in an oven heated at 125 °C for 20 h. After the autoclave was cooled down to room temperature, 9 g of KCl was added to the light-yellow solution and the solution was stirred for 30 min. The produced light-yellow solid was centrifuged under 4400 rpm for 5 mins. Recrystallization was carried by dissolving this solid in 30 mL deionized water (100 °C, heated in an oil bath). White crystals were formed in next few days (normally within three days) and collected by filtration. More pure crystals were obtained by recrystallization one more time from 20mL of deionized

water at 100 °C (heated in an oil bath), and the white crystals were collected by filtration. The structure and isomeric purity were confirmed by ^{31}P NMR (-10.05 ppm in D_2O).

Synthesis of $K_{28}Li_5H_7[P_8W_{48}O_{184}]·92H_2O$: The original synthetic procedure of synthesizing $K_{28}Li_5H_7[P_8W_{48}O_{184}]·92H_2O$ was reported in the literature.[3] However, we have found that the former method has two important obstacles., namely, yield and prolong time of crystallisation. Therefore, Cronin group has developed new synthetic protocol for the preparation of KLi-{P_8W_{48}} derivatives, which was followed for this work. As with the preparation of $K_{12}H_2[\alpha\text{-}P_2W_{12}O_{48}]·24H_2O$, fresh starting material was used to ensure the optimum yield.

Procedure: To 200 mL H_2O in a 250 mL beaker, was added 6.0 mL CH_3CO_2H, followed by lithium acetate dihydrate ($LiCH_3CO_2·2H_2O$, 9.0 g, 88 mmol). After 5 minutes of vigorous stirring to allow the $LiCH_3CO_2$ to dissolve, LiCl (4.24 g, 100 mmol) was then added, before again allowing 5 minutes to dissolve. Finally, $K_{12}H_2[\alpha\text{-}P_2W_{12}O_{48}]$ $24H_2O$ (5.60 g, 1.42 mmol) was added over a period of 30 minutes, whilst maintaining vigorous stirring. Once the addition was complete, the solution was stirred for ten further minutes, before setting-aside in a temperature-controlled room (18°C) for crystallisation. The white colour needle shaped crystals were collected after 7 days and used as such without further purification.

Yield: 750 mg, 51 μmol, 14.3 % based on P/W. The bulk purity of the KLi-{P_8W_{48}} was confirmed by ^{31}P NMR. The major peak was appeared at -7.24 ppm (in LiCl/D_2O).

References SI-1

1 C. Kato, S. Nishihara, R. Tsunashima, Y. Tatewaki, S. Okada, X.-M. Ren, K. Inoue, D.-L. Long, L, Cronin, *Dalton Trans*., **2013**, 42, 11363–11366

2 a) A. Hayashi, T. Haioka, K. Takahashi, B. S. Bassil, U. Kortz, T. Sano, M. Sadakane, *Z. Anorg. Allg. Chem.* **2015**, 641, 2670-2676. b) M. J. Turo, L. Chen, C. E. Moore, A. M. Schimpf, *J. Am. Chem. Soc.* **2019**, 141, 4553-4557.

3 a) R. Contant, A. Tézé, Inorg. Chem., **1985**, 24, 4610-4614; b) R. Contant*, in Inorganic Syntheses*, (Ed. A. P. Ginsberg, John Wiley & Sons, New York, **1990**, pp. 110-111. c) Thomas Boyd, Ph.D. thesis, University of Glasgow, **2014.**

SI-2: Comparison with different counter cation of W-Dawson cluster

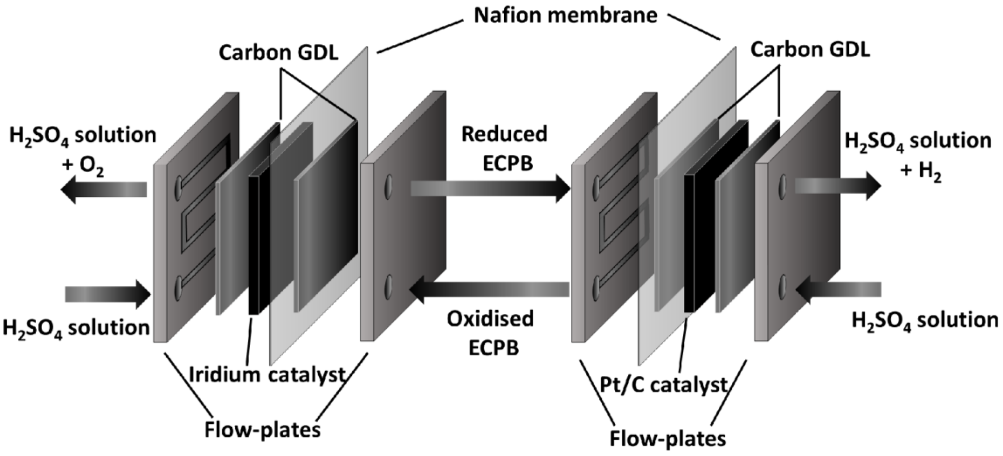

Figure S2-1. Schematic representation of the flow cells system used for the oxidation and reduction of $X_6[P_2W_{18}O_{62}]$ being X=Li, Na and K $X_6[P_2W_{18}O_{62}]$-mediated H_2O splitting with decoupled OER and HER. HER, hydrogen evolution reaction; OER, oxygen evolution reaction. In the case of the reduction-oxidation cycling of 0.05 M $Li_6[P_2W_{18}O_{62}]$ in 1 M H_2SO_4 at a current density of ±50 mA cm^{-2}. All data here were collected at 20 °C using 25 ml 0.05 M of each salt, e.g. $Li_6[P_2W_{18}O_{62}]$ in 1 M H_2SO_4, in the working electrode flow cell part, while 1 M H_2SO_4 was used as the electrolyte for OER and HER reaction counter parts and Hg/$HgSO_4$ (1 M H_2SO_4, 0.68 vs. NHE) was used as reference electrode. This test is done before the degassing with Ar atmosphere for 1 h.

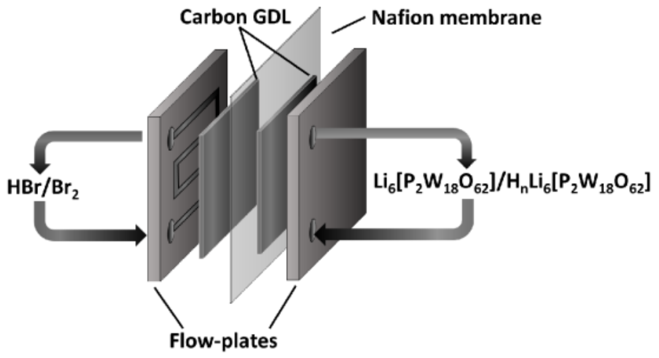

Figure S2-2. Schematic redox flow battery with HBr/Br_2 as positive redox couple and $Li_6[P_2W_{18}O_{62}]$/$H_nLi_6[P_2W_{18}O_{62}]$ as negative redox couple

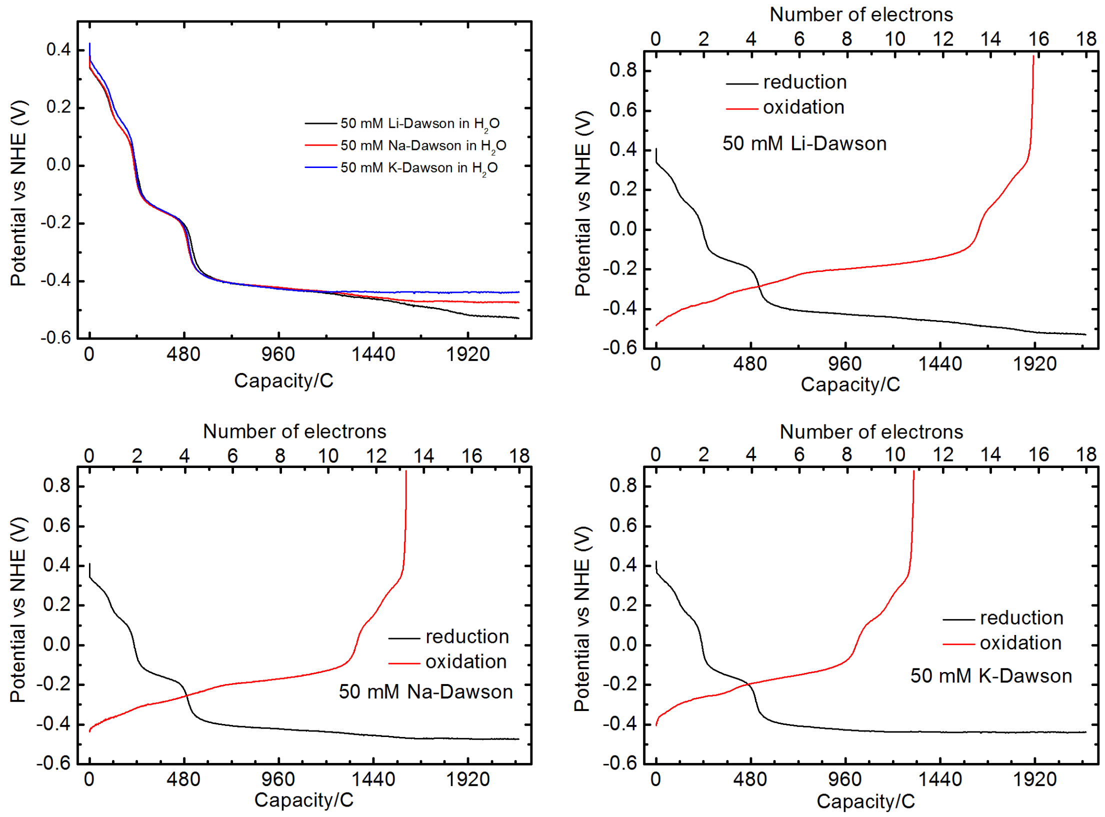

Figure S2-3. The comparison of the redox behaviour of 25ml 50mM $Li_6[P_2W_{18}O_{62}]$, $Na_6[P_2W_{18}O_{62}]$ and $K_6[P_2W_{18}O_{62}]$ in water at a current density of ±50 mA cm^{-2} (±648 mA). As we can see from the above reduction curves, the reduction potentials are a little bit different: $K_6[P_2W_{18}O_{62}]$ > $Na_6[P_2W_{18}O_{62}]$ > $Li_6[P_2W_{18}O_{62}]$. All Dawson clusters undergo similar reduction process in the first 4e- per cluster reduction. And the number of reversible redox electrons is following $Li_6[P_2W_{18}O_{62}]$ > $Na_6[P_2W_{18}O_{62}]$ > $K_6[P_2W_{18}O_{62}]$. This result confirms that the cations have obvious influence on the redox properties of Dawson anion cluster.

SI-3: Electron paramagnetic resonance (EPR)

X-Band EPR spectra were recorded after each reduction step on a Bruker Elexsys E500 spectrometer with a cylindrical TE011 cavity frozen 100 mM $Li_6[P_2W_{18}O_{62}]$ and reduced $Li_6[P_2W_{18}O_{62}]$ with different amount of electrons aqueous solution at T=100K. The number of reduced electrons was controlled by the amount of Coulomb consumed which turns out to control the time, t, since the applied current was constant. For each EPR testing, around 0.1 ml reduced solution was taken out from the cell and frozen by liquid N_2 in the EPR tube. No resonance could be observed for the original 100 mM $Li_6[P_2W_{18}O_{62}]$.

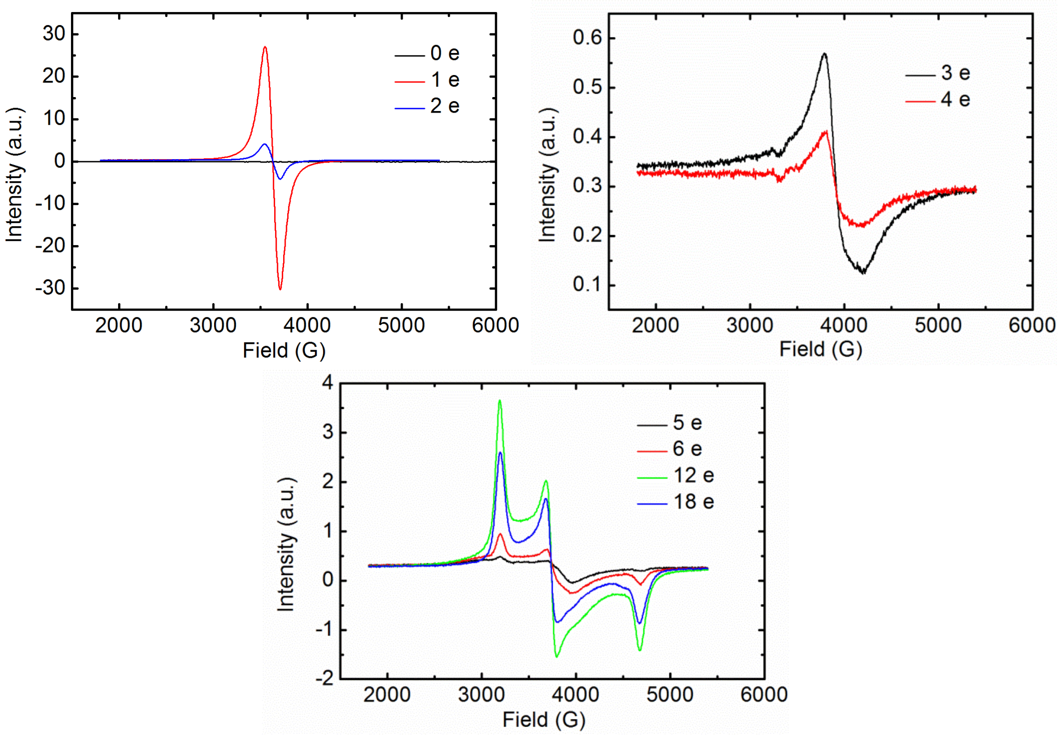

Figure S3-1. EPR results for different number of electrons input to reduce per $Li_6[P_2W_{18}O_{62}]$ cluster. As we can see from the above graphs, the original 100mM $Li_6[P_2W_{18}O_{62}]$ solution shows a very clean background signal which confirms there is no any obvious impurity in the solution.

Geometrically $Li_6[P_2W_{18}O_{62}]$. provides 2 kinds of identical W atoms environment in W-Dawson cluster, including 2 belt W_6 hexagonal belts in between 2 triangular caps W_3O_{13}. In each W_6 belt the octahedral are alternatively sharing edges and corners while the 2 belts are joined by 6 common corners. And the electron paramagnetic resonance (EPR) spectroscopy

results show a g-factor 1.855 EPR resonance can be observed in the 1e reduced sample which corresponds to the 1e- delocalization electrons on one of the internal hexagonal belts.[1] See main text for further discussion of the EPR spectra of frozen solutions of **Li-{P_2W_{18}}-*n*e** (100 mM; *n* = 1, 2, 3, 4, 5, 6, 12, 17).

EPR fitting: The EPR spectra were fitted using PHI 3.1.1 program[2] adopting the following effective spin Hamiltonian:

$$H' = \mu_B(g'_x H_x S'_x + g'_y H_y S'_y + g'_z H_z S'_z)$$

The best fits were obtained using the following parameters: g = 1.856 (**Li-{P_2W_{18}}-1e**); g = 1.857 (**Li-{P_2W_{18}}-2e**); g_x = 2.111, g_y = 1.800, g_z = 1.442 (**Li-{P_2W_{18}}-12e**); g_x = 2.108, g_y = 1.800, g_z = 1.442 (**Li-{P_2W_{18}}-17e**).

References SI-3

1. Varga, G. M.; Papaconstantinou, E.; Pope, M. T. Heteropoly Blues. IV. Spectroscopic and Magnetic Properties of Some Reduced Polytungstates1. *Inorg. Chem.* **1970**, *9* (3), 662–667. https://doi.org/10.1021/ic50085a045

2. Chilton, N. F.; Anderson, R. P.; Turner, L. D.; Soncini, A.; Murray, K. S. PHI: A Powerful New Program for the Analysis of Anisotropic Monomeric and Exchange-Coupled Polynuclear d- and f-Block Complexes. *J. Comput. Chem.* **2013**, *34* (13), 1164–1175. https://doi.org/10.1002/jcc.23234

SI-4: Paramagnetic ^{183}W Nuclear Magnetic Resonance Spectroscopy (^{183}W NMR) of Li-Dawson at different reduced state

^{183}W NMR spectra were recorded in 5 mm o.d. tubes at 25.0 MHz on a Bruker Ascend Aeon 600 MHz NMR spectrometer with a BBO probe head. Chemical shifts are referenced to 2M Na_2WO_4 solution (δ=0 ppm) according to the IUPAC recommendation. High quality spectra, with good signal-to-noise ratio to observe tungsten satellites, required 1 day of spectrometer time, or until a spectrum of suitable resolution was obtained.

Table S4-1-NMR. Summary of ^{183}W data for $Li_6[P_2W_{18}O_{62}]$ Wells-Dawson compound.

Cation	W (cap) / ppm	W (belt) /ppm
Li (measured)	-126	-171
NH_4 (Ref 1)	-123	-168
Na (Ref 2)	-128	-174
Na (measured)	-128	-173

Li-Dawson concentration	W (cap) / ppm	W (belt) /ppm
saturated	-125.7	-171.0
500mM	-127.0	-172.5
50mM	-125.7	-171.0

0.1 M Li-Dawson in C_M (H_2SO_4)	W (cap) / ppm	W (belt) /ppm
Chemical shift	-125.7	-171.0

References SI-4:

1. Boyd, Thomas (**2013**) *From molecular assemblies to extended frameworks of polyoxometalates through secondary metal ion coordination*. PhD thesis, University of Glasgow.

2. R. Acerete, C. F. Hammer, L. C. W. Baker, Tungsten-183 NMR of heteropoly and isopolytungstates. Explanations of chemical shifts and band assignments and theoretical considerations**.** *J. Am. Chem. Soc.* **1982**, 104, 5384 –5390. https://doi.org/10.1021/ja00384a023

SI-5: superconducting quantum interference device (SQUID) data

The magnetic susceptibility of **Li-{P_2W_{18}}-0e, Li-{P_2W_{18}}-1e, Li-{P_2W_{18}}-9e and Li-{P_2W_{18}}-17e** were studied by measuring direct current (dc) magnetic measurements in MPMS-XL SQUID (Quantum Design). As all studied POMs were in liquid state prior to the measurements a precise aliquot of POM 0.1 M solution was transferred under Ar flow into a quartz container (consisting of 5 mm diameter quartz ampoule). The container was then flame sealed with a blow torch.

Prior the measurements of POMs the magnetisation of the quartz container was measured (Fig. S5-1, green trace) in a magnetic field of 1000 Oe. In the higher temperature range the M vs. T data show weak signal consistent with temperature independent diamagnetism expected from quartz. However, at low temperatures there is an evidence for contribution from paramagnetic impurities. Even though spectroscopy-quality quartz ampoule was used as a container, the tiny magnetic impurities are inevitable. In a separate experiment, an aliquot of water was transferred into the quartz container and flame sealed. The magnetisation data confirms diamagnetic properties along the entire 10 – 250 K range in a magnetic field of 1000 Oe. However, there is a deviation from temperature independent magnetism at low temperatures (Fig. S5-2, red trace). By subtracting the contribution of the container from the data, it was possible to evaluate the magnetisation of water (or more precisely ice) between 10 and 250 K (Fig. S5-2, blue trace). It is evident that only within the temperature range of 100 – 250 K it shows temperature independent magnetism therefore, this range was deemed as the most suitable for evaluation of magnetic properties of POMs (Fig. S6-3). The magnetic susceptibilities of POMs were evaluated by subtracting the contribution from container and water from the corresponding signal of the sample after the measurements.

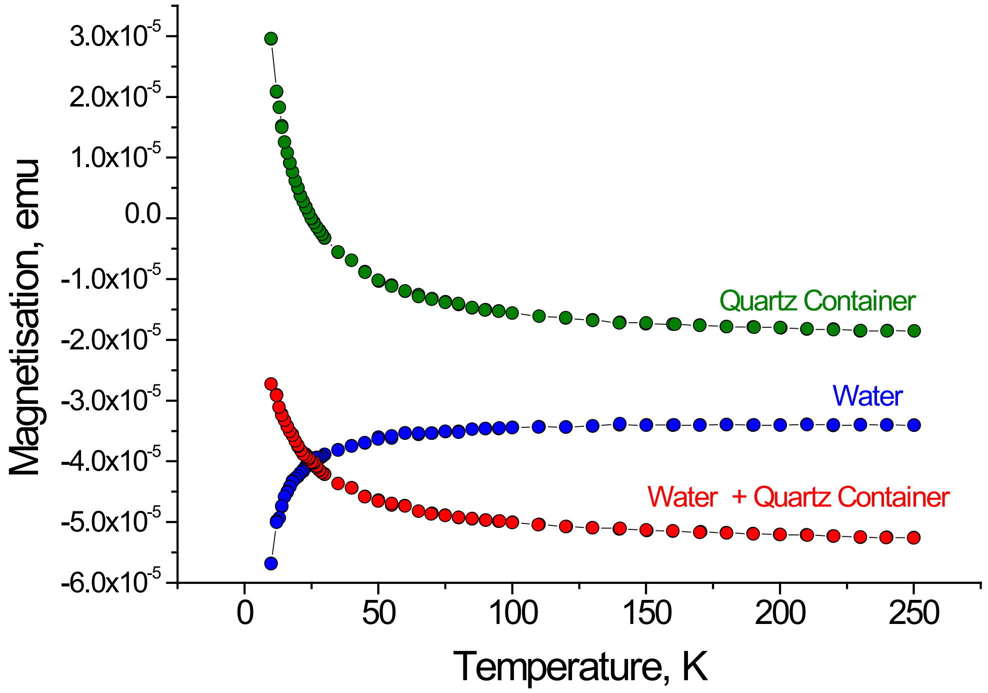

Figure S5-1. The dc-magnetisation of quartz container (green circles), 0.056 ml of deionised water in the quartz container (red circles) and the magnetisation of 0.056 ml of water without contribution from the container in the temperature range 10 – 250 K in a magnetic field of 1000 Oe.

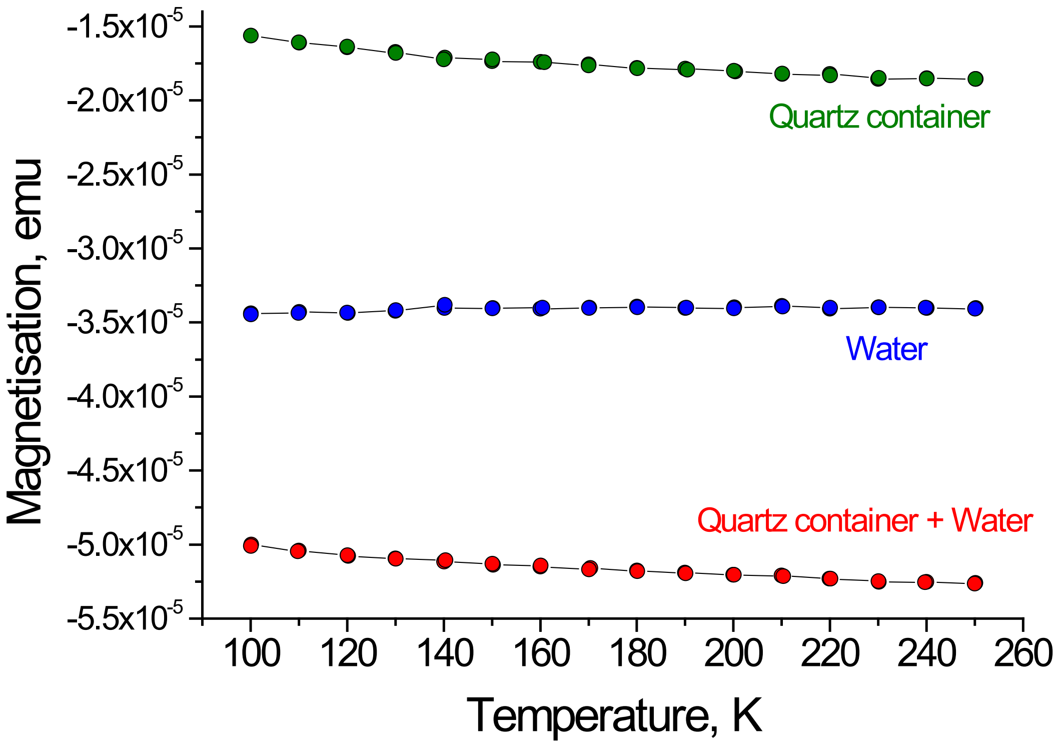

Figure S5-2. The dc-magnetisation of quartz container (green circles); 0.056 ml of deionised water in the quartz container (red circles) and the magnetisation of 0.056 ml of water without contribution from the container in the temperature range 100 – 250 K in a magnetic field of 1000 Oe. The range is consistent with nearly temperature independent magnetism of both water and quartz container.

The molar susceptibility (χ) of **Li-{P_2W_{18}}-0e, Li-{P_2W_{18}}-1e, Li-{P_2W_{18}}-9e and Li-{P_2W_{18}}-17e** is plotted in Fig. Sx. It is evident that with exception of **Li-{P_2W_{18}}-1e** which is paramagnetic with an estimated magnetic moment of *ca*. 1.6 m$_B$ (Fig. S5-2) all studied compounds are diamagnetic. Both **Li-{P_2W_{18}}-9e and Li-{P_2W_{18}}-17e** show very weak magnetism which is practically temperature independent and therefore, one can hypothesise that their behaviour is consistent with metals. (See S6-3 and S6-4 for plots)

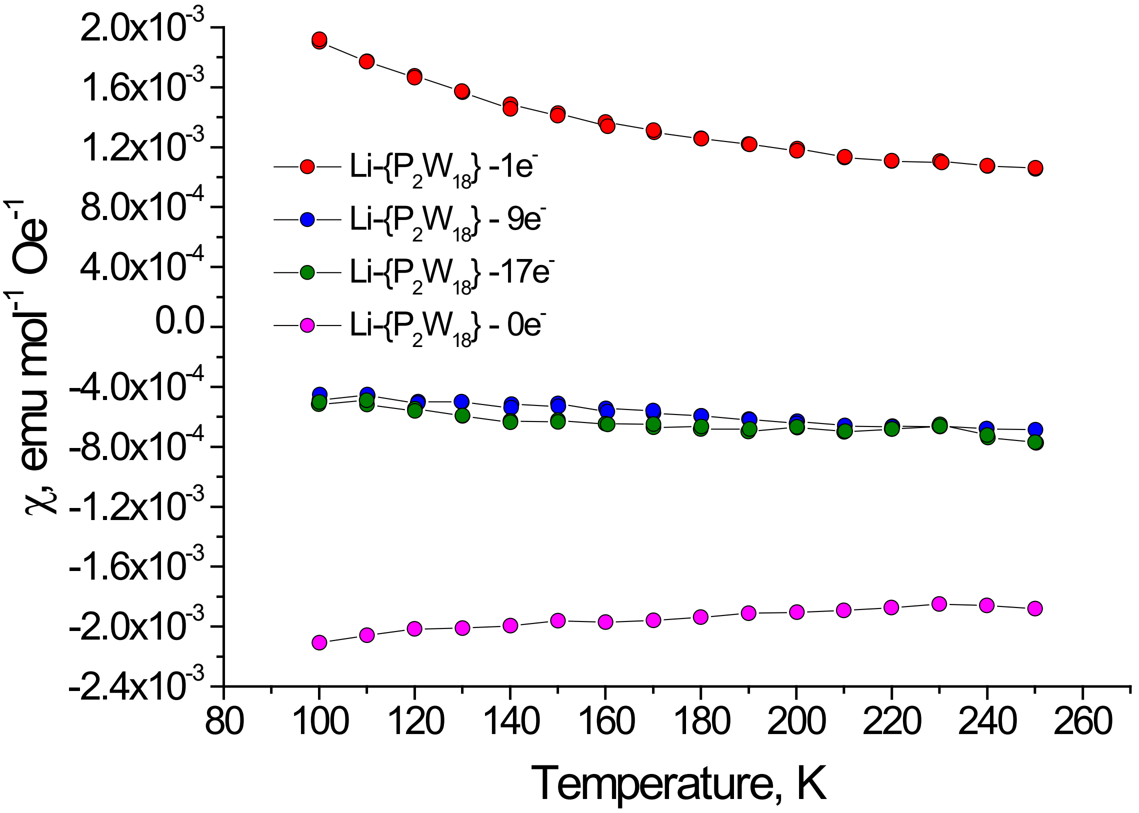

Figure S5-3. The molar susceptibility of **Li-{P_2W_{18}}-0e** (5.68 10^{-6} mol)**, Li-{P_2W_{18}}-1e** (5.85 10^{-6} mol)**, Li-{P_2W_{18}}-9e** (5.74 10^{-6} mol) **and Li-{P_2W_{18}}-17e** (5.53 10^{-6} mol) in the temperature range of 100 – 250 K in a magnetic field of 1000 Oe. The data is corrected for contributions from water and quartz container.

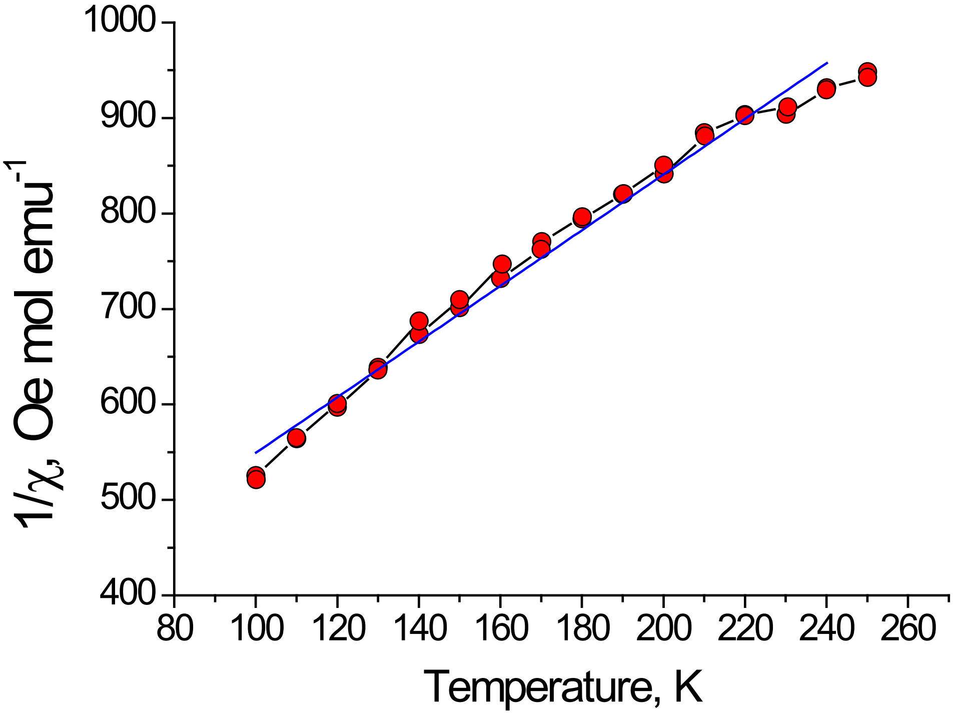

Figure S5-4. The inverse molar susceptibility of **Li-{P_2W_{18}}-1e** (5.85 10^{-6} mol) in the temperature range of 100 – 250 K (in a magnetic field of 1000 G) fitted with a linear plot with the slope of 2.91. The data is corrected for contributions from water and quartz container.

SI-6: Small-angle X-ray scattering (SAXS) data

Benchtop X-ray scattering data were collected on an Anton Paar SAXSess instrument using Cu-Kα radiation (1.54 Å) and line collimation. The instrument was equipped with a 2-D image plate for data collection in the q = 0.018-2.5 $Å^{-1}$ range. The lower q resolution is limited by the beam attenuator. Approximately 20 mmolar solutions were measured in 1.5 mm glass capillaries (Hampton Research). Scattering data of neat solvent was collected for background subtraction. Scattering was measured for 30 min for every experiment. We used SAXSQUANT software for data collection and treatment (normalization, primary beam removal, background subtraction, desmearing, and smoothing to remove the extra noise created by the desmearing routine). All analyses and curve-fitting to determine R_g, size, shape and size distribution were carried out utilizing IRENA macros with IgorPro 6.3 (Wavemetrics) software.[1] To simulate scattering data of P_2W_{18} monomer and dimer aggregates, we used SolX software.[2]

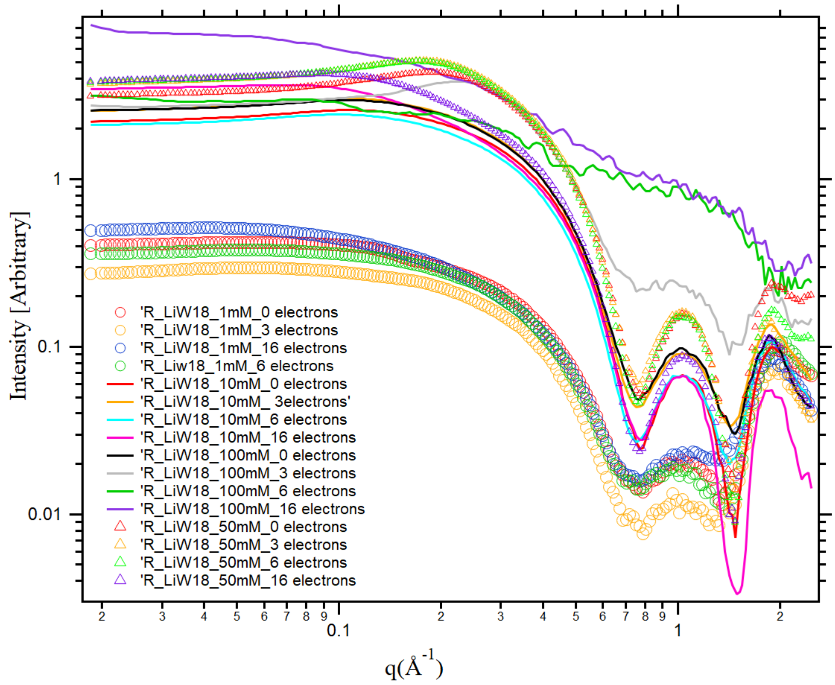

Figure S6-1-SAX. SAXS of all **X-Li-P_2W_{18}-X** samples (intensity not normalized).

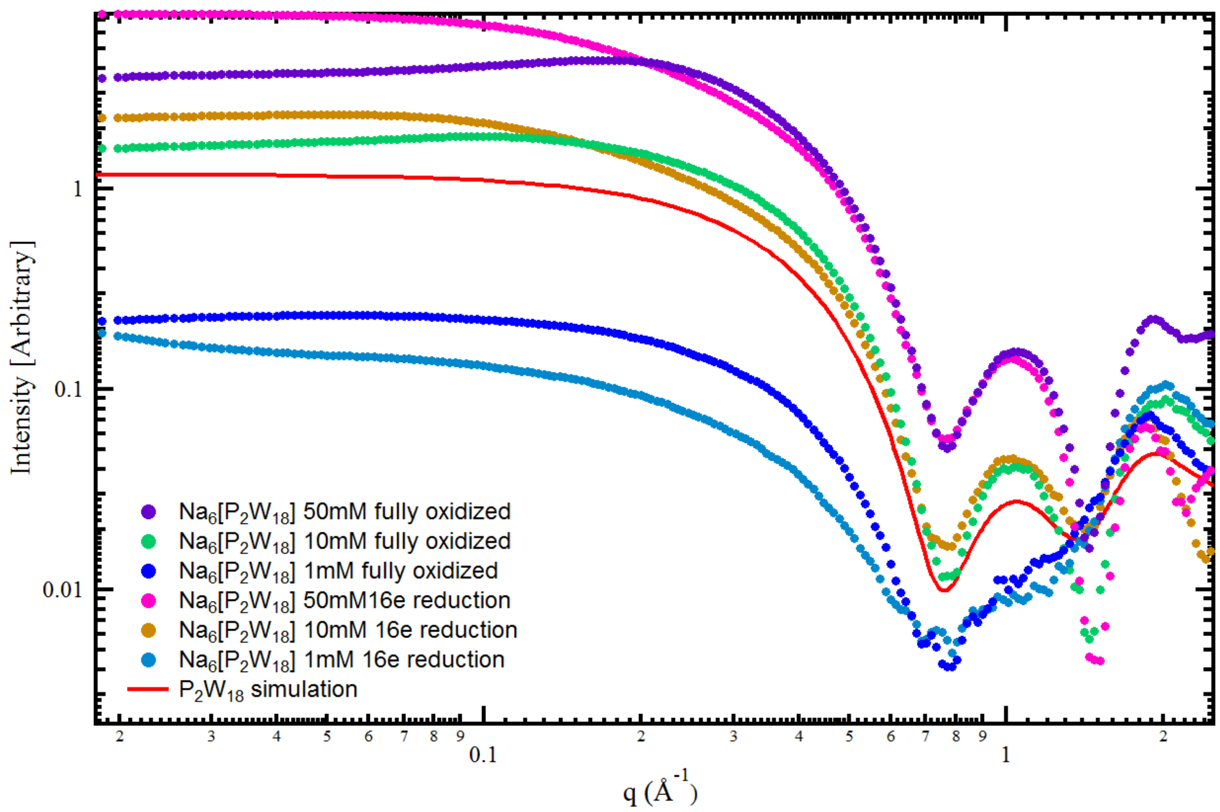

Figure S6-2-SAX. SAXS of all **X-Na-P_2W_{18}-X** samples (intensity not normalized).

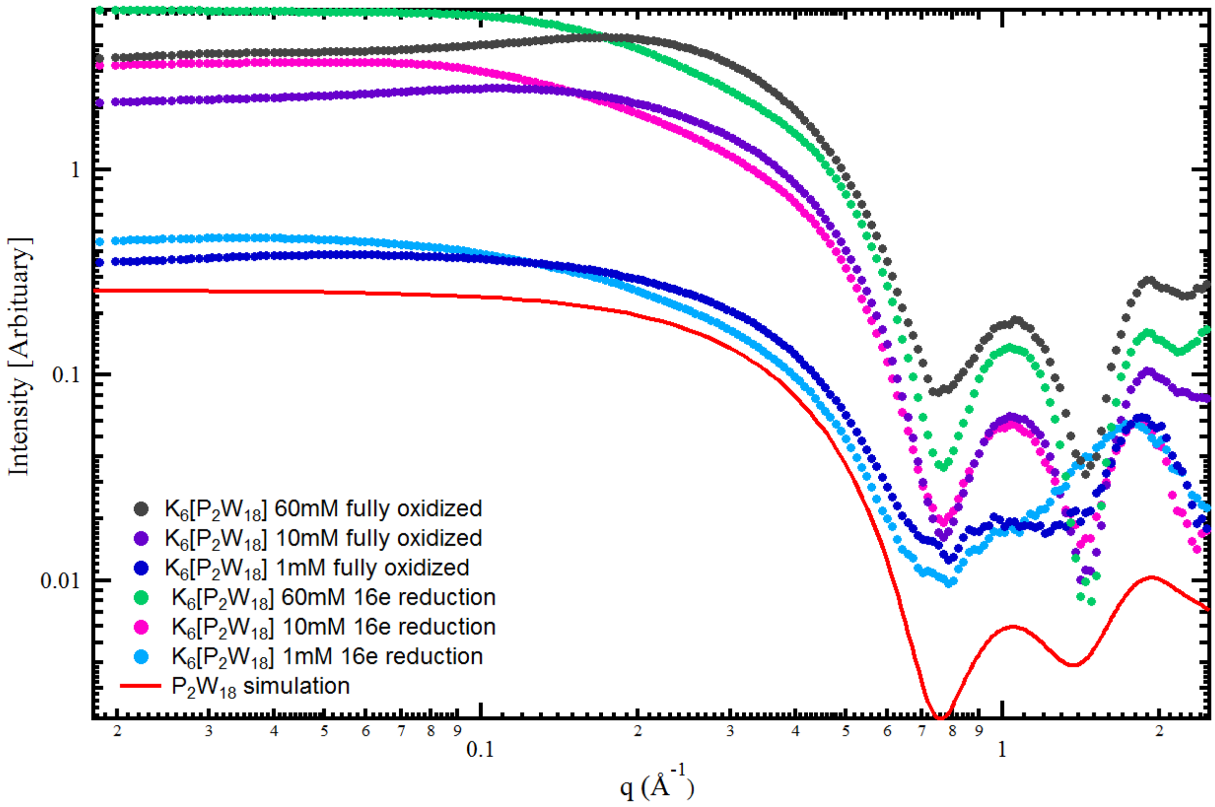

Figure S6-3-SAX. SAXS of all **X-K-P_2W_{18}-X** samples (intensity not normalized).

Table S6-1-SAXS. Summary of X-A-P_2W_{18}-Y samples analyzed by SAXS[1]

Electron count	Li				Na				K		
**0e$^-$**	**1-Li-P_2W_{18}-0**	**10-Li-P_2W_{18}-0**	**50-Li-P_2W_{18}-0**	**100-Li-P_2W_{18}-0**	**1-Na-P_2W_{18}-0**	**10-Na-P_2W_{18}-0**	**50-Na-P_2W_{18}-0**	**100-Na-P_2W_{18}-0**	**1-K-P_2W_{18}-0**	**10-K-P_2W_{18}-0**	**60-K-P_2W_{18}-0**
**3e$^-$**	**1-Li-P_2W_{18}-3**	**10-Li-P_2W_{18}-3**	**50-Li-P_2W_{18}-3**	**100-Li-P_2W_{18}-3**							
**6e$^-$**	**1-Li-P_2W_{18}-6**	**10-Li-P_2W_{18}-6**	**50-Li-P_2W_{18}-6**	**100-Li-P_2W_{18}-6**							
**18e$^-$**	**1-Li-P_2W_{18}-18**	**10-Li-P_2W_{18}-18**	**50-Li-P_2W_{18}-18**	**100-Li-P_2W_{18}-18**	**1-Na-P_2W_{18}-18**	**10-Na-P_2W_{18}-18**	**50-Na-P_2W_{18}-18**	**100-Na-P_2W_{18}-18**	**1-K-P_2W_{18}-18**	**10-K-P_2W_{18}-18**	**60-K-P_2W_{18}-18**

[1](X=concentration in millimolar, A=alkali, Y=number of electrons for the reduced samples)

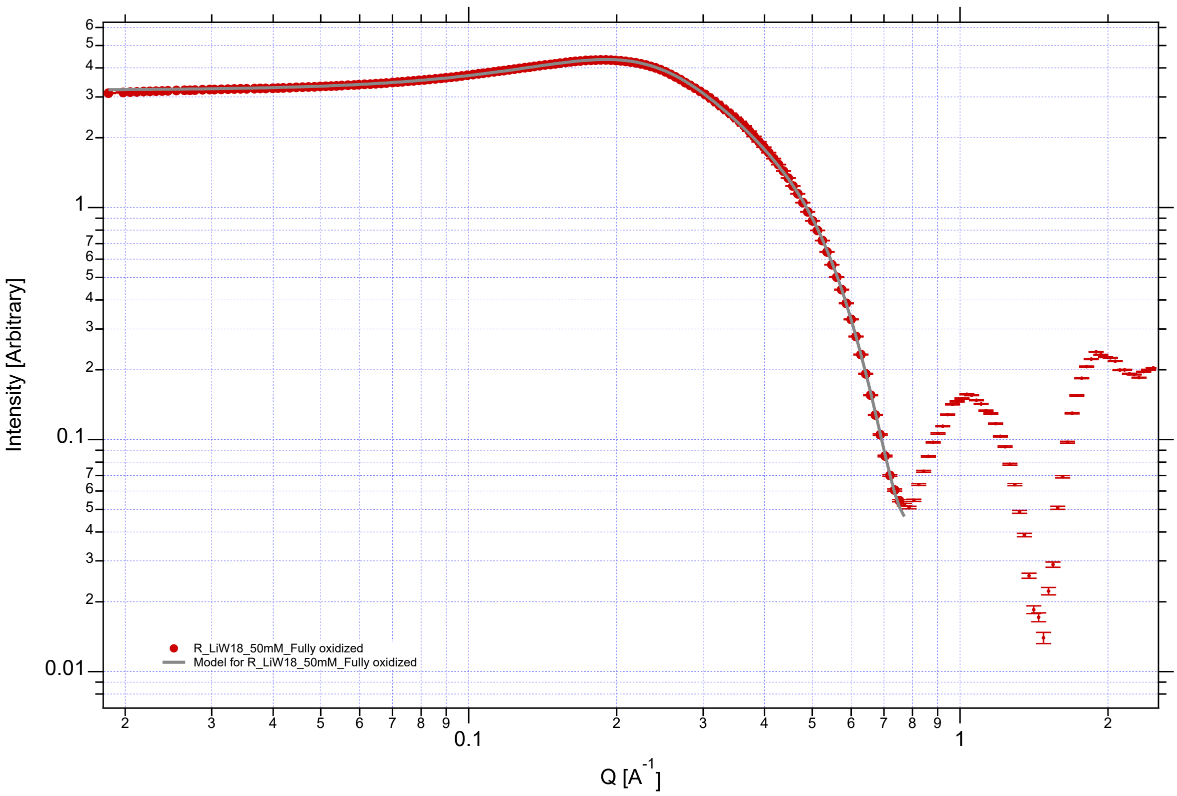

Figure S6-4-SAXS. Fitting the 50-Li-{P_2W_{18}} (fully oxidized) scattering curve with form and structure factors (see Table 1 in manuscript for fitting parameters. Red markers is experimental data (plus error bars), gray line is the model fit of the scattering data.

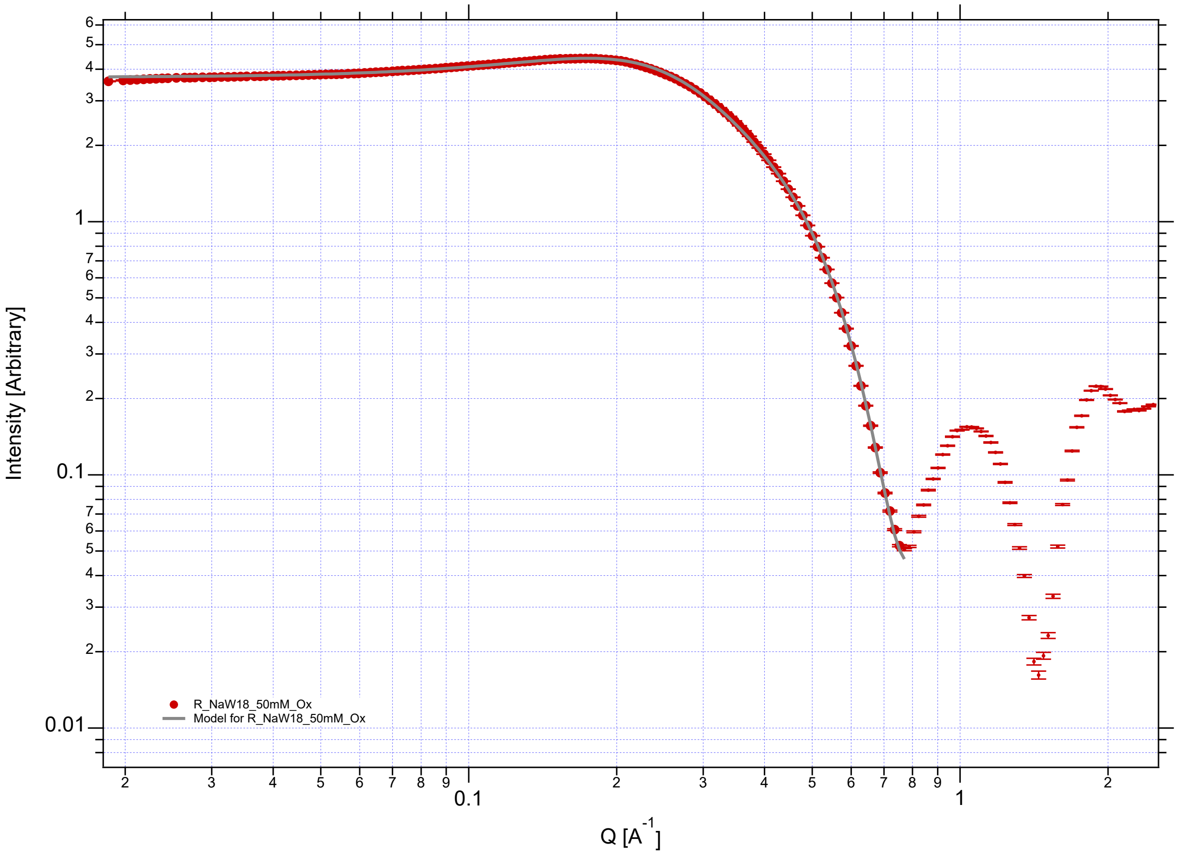

Figure S6-5-SAXS. Fitting the 50-Na-{P_2W_{18}} (fully oxidized) scattering curve with form and structure factors (see Table 1 in manuscript for fitting parameters. Red markers is experimental data (plus error bars), gray line is the model fit of the scattering data.

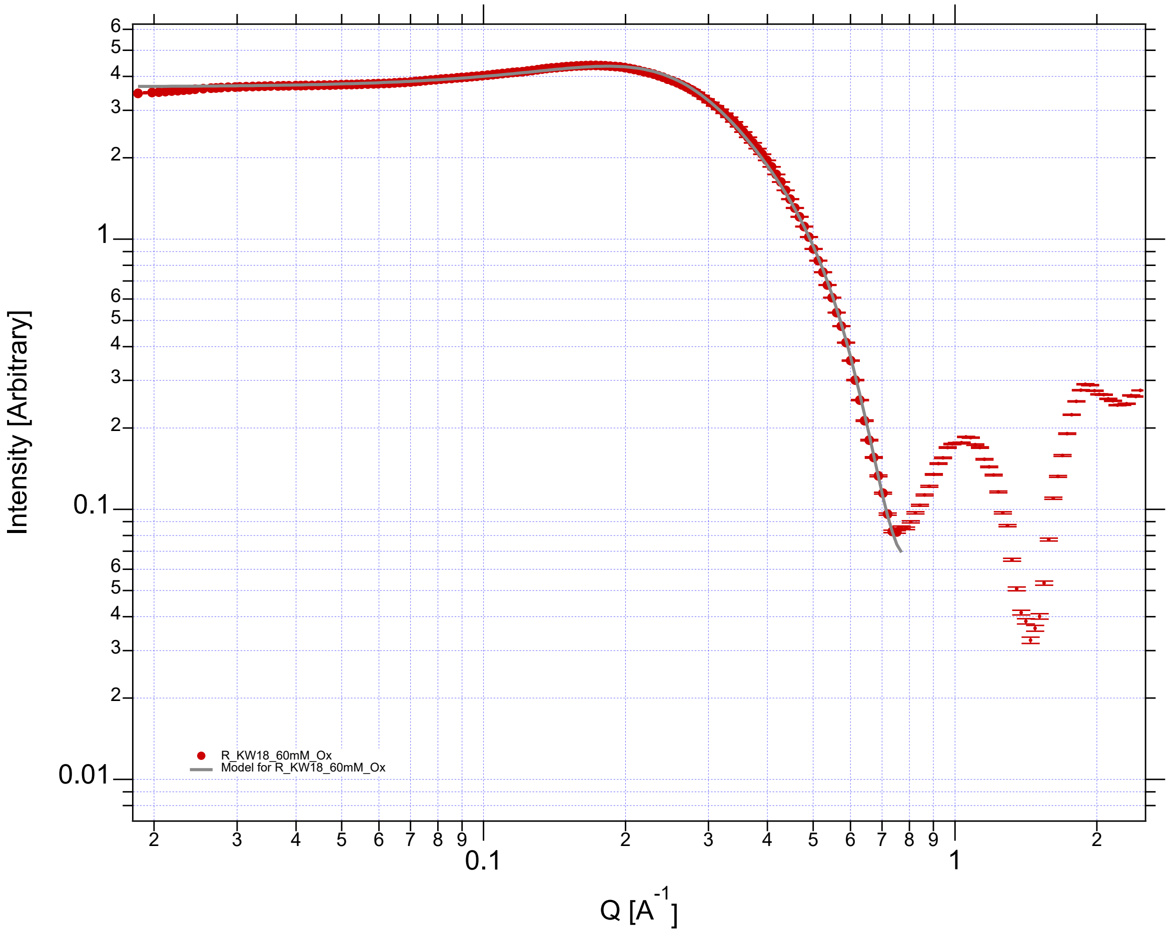

Figure S6-6-SAXS. Fitting the 60-K-{P_2W_{18}} (fully oxidized) scattering curve with form and structure factors (see Table 1 in manuscript for fitting parameters. Red markers is experimental data (plus error bars), gray line is the model fit of the scattering data.

References SI-6

1. Ilavsky, J.; Jemian, P. R. ıt Irena: tool suite for modeling and analysis of small-angle scattering. *Journal of Applied Crystallography* **2009**, *42* (2), 347–353.

2. Zuo, X.; Cui, G.; Merz, K. M.; Zhang, L.; Lewis, F. D.; Tiede, D. M. X-ray diffraction “fingerprinting” of DNA structure in solution for quantitative evaluation of molecular dynamics simulation. *Proceedings of the National Academy of Sciences of the United States of America* **2006**, *103* (10), 3534—3539.

SI-7: UV-vis device

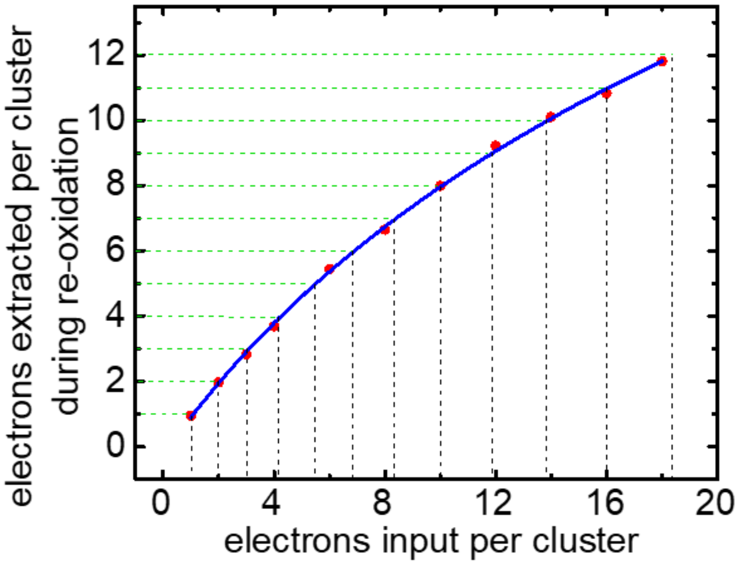

Figure S7-1. From this map, the exact number of stable electrons in the 10mM $Li_6[P_2W_{18}O_{62}]$ solution can be prepared by input the required electrons to reduce it. For example, to get a exact number of 7 electrons, a number of 8.34 electrons is needed. Then we just need to pass the equivalent amount of charge of 8.34 e to reduce the solution

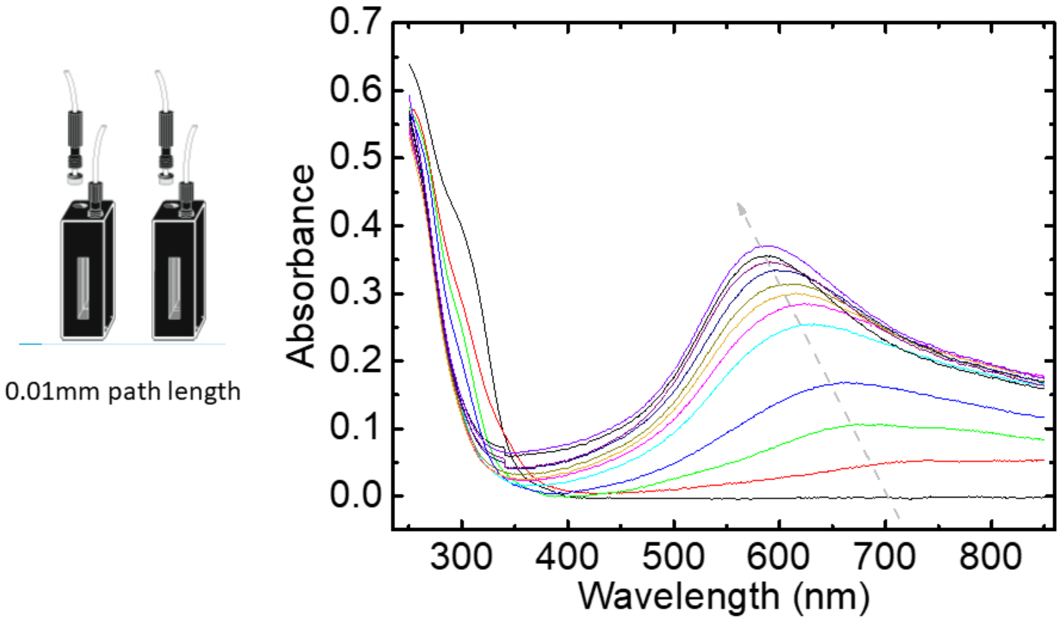

Figure S7-2. Thin layer device to obtain 10mM $Li_6[P_2W_{18}O_{62}]$ UV-vis results of 10mM Li-Dawson solution at various reduction states: from 0 e to 12 e The solution can be prepared by input the required electrons to reduce it. The band in spectrum at 550-650 nm probes the population of W-based MOs. Observation of a shift to more energetic transitions and the disappearance of the and at ca. 300 nm

Aiming to explain the evolution of experimental UV-Vis spectrum of **{P_2W_{18}}** upon reduction, we simulated the UV-Vis spectra of the cluster with 0, 2 and 6 electrons. These spectra are compared in Figure S7-3. As in the experimental spectrum, we observed that the band at *ca.* 300 nm in the spectrum of the fully-oxidised species associated to p(O)→d(W) transitions decreases its intensity in the spectrum of the 2 electron-reduced one and completely disappears after further reducing the system. This is caused by the effect of populating the lowest d(W) orbitals, preventing the transitions from the oxo band to these orbitals. Also, we observed the appearance of a band centred at *ca.* 600 nm in **{P_2W_{18}}-2e**, which is associated to d(W)→d(W) transitions. Also in agreement with the experimental data, this band is shifted to more energetic transitions when the system is further reduced. The main transition in **{P_2W_{18}}-2e** occurs from the HOMO to the LUMO+6 (ΔE = 3.61 eV). The equivalent transition was identified as the most intense one for **{P_2W_{18}}-6e** (from HOMO-2 to LUMO+4), but since the higher overall charge shifts up the empty orbitals, it is more energetic (ΔE = 4.16 eV). In addition, the band in the spectrum of **{P_2W_{18}}-6e** has also important contribution of other highly energetic transitions such as HOMO→LUMO+18 (ΔE = 4.00 eV). For this reason, the d(W)→d(W) band in highly reduced anions appears at lower values of wave number than in others with less metallic electrons.

As **{P_2W_{18}}-6e** is likely to be protonated at experimental conditions, we also evaluated the effect of having associated protons by computing the spectrum for the **H_3{P_2W_{18}}-6e**, which might represent a sensible model for the protonation state of the cluster at the experimental conditions (*see section 1*). Comparison between the absorbance of **{P_2W_{18}}-6e** and **H_3{P_2W_{18}}-6e** (Figure S7-4) suggests that protonation of the cluster might broaden the bands due to geometrical distortion of the structure. However, it has to be considered that protons are itinerant all over the structure instead of being located on three single sites. This effect should

average out the local distortions resulting in a spectrum more alike to that computed for the bare cluster.

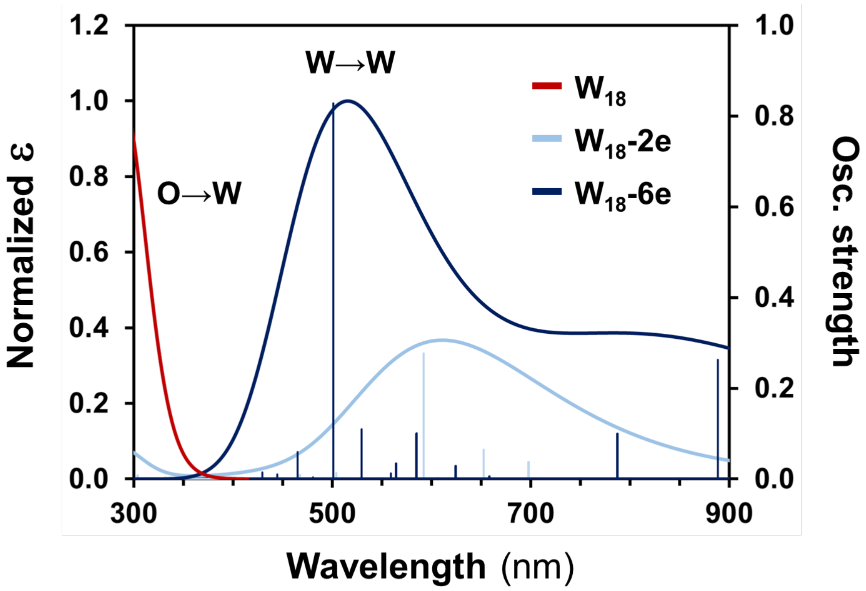

Figure S7-3 Computed UV-Vis spectra for the fully oxidized **$\{P_2W_{18}\}$** anion (red line) and the 2 and 6 electron-reduced **$\{P_2W_{18}\}$-2e** and **$\{P_2W_{18}\}$-6e** (light and dark blue lines, respectively).

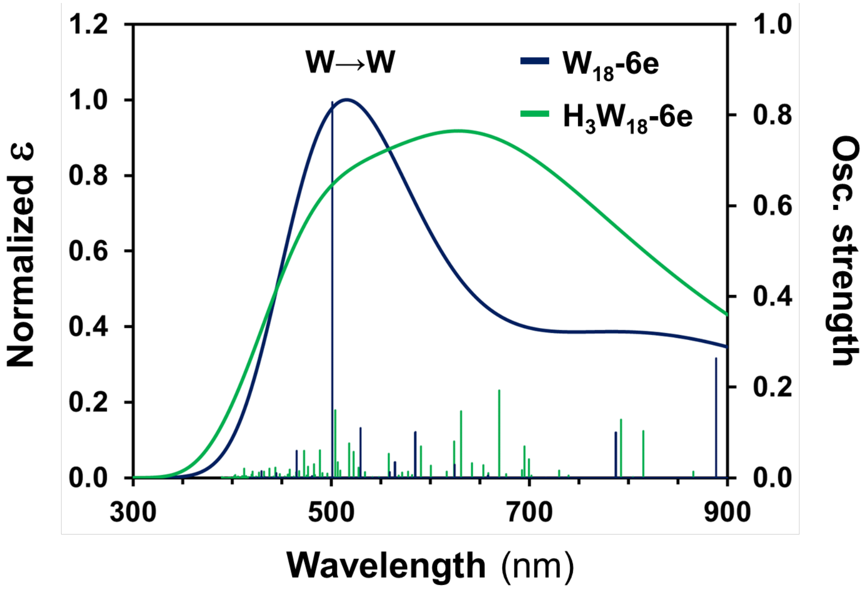

Figure S7-4 Comparison between the UV-Vis spectra of **$\{P_2W_{18}\}$-6e** (solid line) and its protonated counterpart **$H_3\{P_2W_{18}\}$-6e** (dashed line).

SI-8 Theoretical analysis of the super-reduced Wells-Dawson polyoxotungstate.

1. Electronic structure of Dawson anion and theoretical analysis of initial reduction states

Using a GGA functional (BP86) and Slater TZP basis set, we have computed the redox potentials with respect to NHE. Taking an absolute free energy of -4.24 eV for NHE,[1] the redox potential for the first reduction was found to be -0.04 V, which is about 200 mV more negative than the observed potential for the Dawson anion. In terms of molecular orbital energies and neglecting the bielectronic effects, this would mean that the energy of the LUMO should be somewhat more negative than what we get. In line with this relatively small deviation, when we compare experimental and theoretical redox potentials we reproduce rather well the trends for two successive electron reductions, as shown in Table S8-1. Hence, the calculated values of 269, 470 and 217 mV show good correspondence with the experimental ones of 180, 390 and 190 mV, although the computed values are slightly shifted towards more negative potentials.

In acidic conditions (pH 4) the cyclic voltammograms also show four reversible waves, the two first being almost identical to the observed in neutral solution, but the third and fourth correspond to two-electron reductions. Given that the third wave is centered at -0.24 V, very close to the value of -0.22 V observed for the incorporation of the third electron in the neutral solution, we can assume that the three following processes occur: i) the reduction of the **{P_2W_{18}}-2e** species, ii) the chemical protonation of **{P_2W_{18}}-3e** induced by the increase of the anion basicity, and iii) reduction of the protonated anion to give **H{P_2W_{18}}-4e**. Indeed, DFT calculations show that the protonation shifts the redox potential for three-electron reduced species in more than 120 mV toward more positive values. This value was estimated protonating only some of the most basic bridging oxo sites, however the protons are most likely itinerant among several sites and this could somewhat modify the effect of protonation on the

redox potentials. It is expected that further reductions should occur via successive protonation/electron-reduction steps to give **$H_2\{P_2W_{18}\}$-4e**, **$H_2\{P_2W_{18}\}$-5e**, **$H_3\{P_2W_{18}\}$-5e**, **$H_3\{P_2W_{18}\}$-6e**, etc. As described in the main text, POM agglomeration phenomena also play an important role in the stability and redox properties of the polyoxotungstates.

Table S8-1. Comparison of Experimental and Computed Reduction Potentials for $[P_2W_{18}O_{62}]^{6-}$ at pH=7.

	**W_{18} → W_{18}-1e**	**W_{18}-1e → W_{18}-2e**	**W_{18}-2e → W_{18}-3e**	**W_{18}-3e → W_{18}-4e**
E^{red} a)	+0.35	+0.17	-0.22	-0.40
ΔE^{red} (expt)b)		-190	-390	-180
ΔE^{red} (DFT) b)		-217	-470	-269

a) Reduction potentials in V vs SHE; b) reduction potential differences for two successive electron reductions in mV. All the reduction potentials were computed with a BP86 functional and a Slater TZP basis set.

To further prove that reduced $\{P_2W_{18}O_{62}\}$ clusters are prone to associate protons to its framework at highly acidic conditions, we carried out a DFT-MD simulation for the **$\{P_2W_{18}\}$-4e** species. Previous to this, we performed an initial classical MD simulation of **$\{P_2W_{18}\}$-4e** at pH $\approx$ 1 that showed that hydronium cations tend to accumulate close to the highly negatively charged anion (q = 10-) (see Figure S8-1). Thus, we started the DFT-MD simulation from a configuration in which the POM is surrounded by several H_3O^+ cations that are hydrogen-bonded to its terminal oxo groups. Figure S8-2 shows the evolution of the number of protons associated to the cluster during 5.5 ps of simulation. At t = 0.5 ps the system has already taken 3 protons at terminal oxo sites and after more than 2 ps oscillating between geometries with a number of protons ranging from 2 to 5, the system mostly remains *tri-protonated* until the end of the simulation.

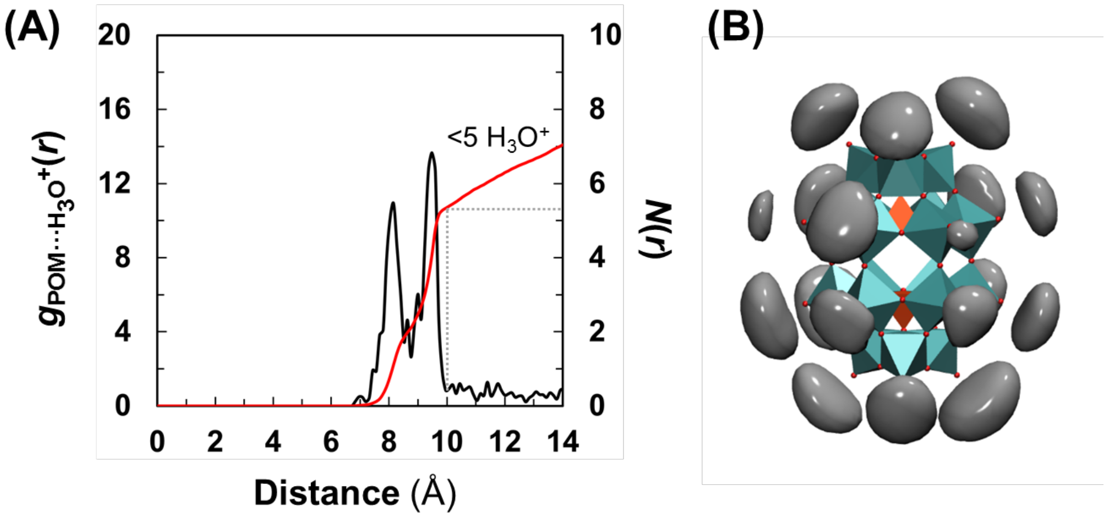

Figure S8-1: (A) Radial Distribution Function (RDF) between $\mathbf{W_{18}}$**-4e** anion and H_3O^+ cations taking as a reference the centre of mass (COM) of both species. Black line represents the RDF itself while the red one represents the integration of the RDF, *N*(*r*), i.e. the average number of H_3O^+ found within the COM of the POM and every distance. First and second peaks are associated to contacts at the belt and the cap regions of the anion, respectively. The distance corresponding to the first minimum after the second peak integrates to 5.3 H_3O^+ cations. This indicates that at least 5 H_3O^+ ions are hydrogen-bonded to the POM in every frame of the simulation. (B) The volumetric density of H_3O^+ (grey volumes) identifies the regions around the terminal oxygen atoms of the POM as those with the highest density of H_3O^+. Data sampled every 2 ps along a 5 ns MD run.

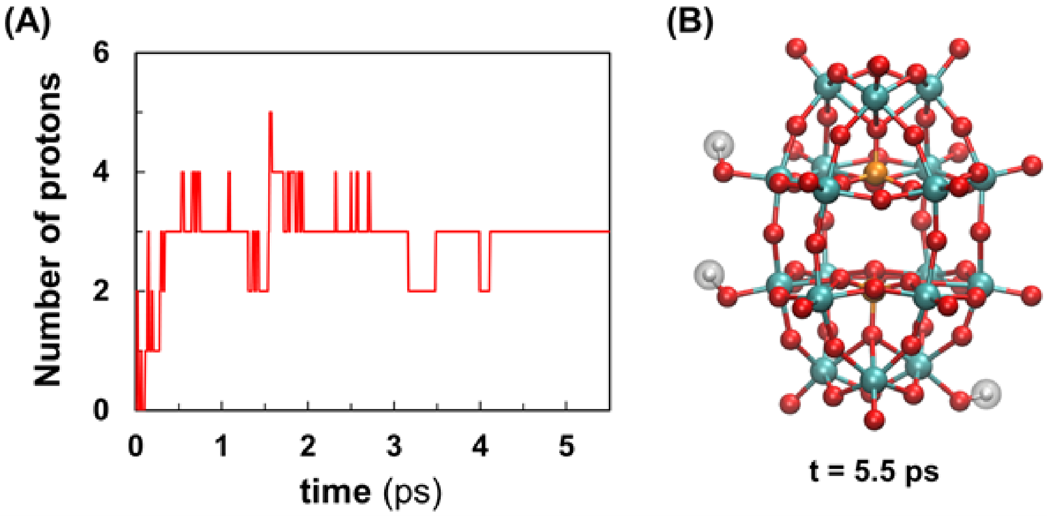

Figure S8-2: (A) Evolution of the number of protons associated to $\mathbf{\{P_2W_{18}\}}$**-4e** during a 5.5 ps DFT-MD simulation. A distance threshold of 1.3 Å was considered for an O—H bond. (B) Snapshot of the protonated cluster ($\mathbf{H_3\{P_2W_{18}\}}$**-4e** , q = 7-) at the end of the simulation, highlighting the three protons taken by the bare anion.

If longer simulation times were computationally affordable for such big systems (<800 atoms), we may observe the diffusion of these associated protons from the most accessible terminal positions to the bridging ones that are more basic.[2] In fact, static DFT calculations indicate that moving 2 protons to bridging positions and releasing the third one to give **$H_2\{P_2W_{18}\}$-4e** species would stabilize the cluster by about 28 kcal mol^{-1} in terms of Gibbs free energy. In **$H_2\{P_2W_{18}\}$-4e**, adding a proton at a terminal position is endothermic by 8.4 kcal mol^{-1}. Releasing another proton to give **$H\{P_2W_{18}\}$-4e** is also endothermic although to a lower extent (4.5 kcal mol^{-1}). Thus, it might be reasonable to think that at pH close to 1 **W_{18}-4e** is di- or mono-protonated. From this point on and assuming a proton-coupled nature for every following reduction process, it might be sensible to expect a number of protons close to 16 in the structure of the 18 electron-reduced system.

2. Polyoxometalate aggregation as a function of the reduction state.

First, 40 ns of classical MD simulations with 50 [**$H\{P_2W_{18}\}$−4e**]$^{9-}$, 50 [**$H_2\{P_2W_{18}\}$−4e**]$^{8-}$ or 50 [**$H_3\{P_2W_{18}\}$−6e**]$^{9-}$ anions at the experimental conditions: POM concentration of 100 mM with 300 Li^+ counter cations and counterbalancing the charge with H_3O^+, obtaining a pH close to 1. These simulations were compared to that with 50 fully-oxidized **$\{P_2W_{18}\}$**$^{6-}$anions in similar conditions.

Visual analysis of the computed trajectories revealed a rather different behavior between the oxidized and reduced POMs. While **$\{P_2W_{18}\}$** anions remain as monomers during the whole simulation, the reduced ones tend to form agglomerate structures. These differences can be appreciated from the comparison of the POM···POM Radial Distribution Functions (RDFs) averaged over the last 10 ns of each simulations, which are depicted in Figure S8-3A. The **$\{P_2W_{18}\}$···$\{P_2W_{18}\}$** RDF does not show any peak, as a proof for a completely lack of agglomeration. However, those for reduced POMs display an array of peaks between 12.6 and

18.9 Å, indicating that there is a set of preferred intermolecular distances between reduced anions to interact. Within this range of distances, the POM anions were found to interact mainly via *lithium-mediated* contacts but also involving hydronium cations acting as linkers and direct H-bonds. The most representative interaction modes between anions are collected in Figure S8-4. Both **H{P_2W_{18}}−4e** and **H_3{P_2W_{18}}−6e** (with an overall charge of 9-) tend to agglomerate into large *lithium-mediated* oligomers similarly. The aggregative behaviour of **H{P_2W_{18}}−4e** and **H_3{P_2W_{18}}−6e** anions arises from their high charge density at the surface. This fact renders their terminal oxygens charged enough to partially remove the solvation shell from the highly hydrophilic cations providing longer-lived direct contacts. Interestingly, the lower charged **H_2{P_2W_{18}}−4e** (q = 8-) anions only present incipient H-bonded adducts. Most likely, this lower ability to agglomerate is due to the fact that **H_2{P_2W_{18}}−4e** anions are not charged enough to pull the solvation shell out of lithium cations so efficiently. Figure S8-3 also compares the POM···Li^+ RDFs for the simulated anions. The same occurs with the fully oxidized **{P_2W_{18}}** cluster, but as it is not basic enough to have associated protons, there cannot be direct H-bond contacts between anions. In fact, a similar trend was observed when analyzing the influence of the charge on the ability to agglomerate of mixed-valence polyoxovanadate clusters, since only the highest charged ones showed ability to agglomerate with alkali metal cations.[3]

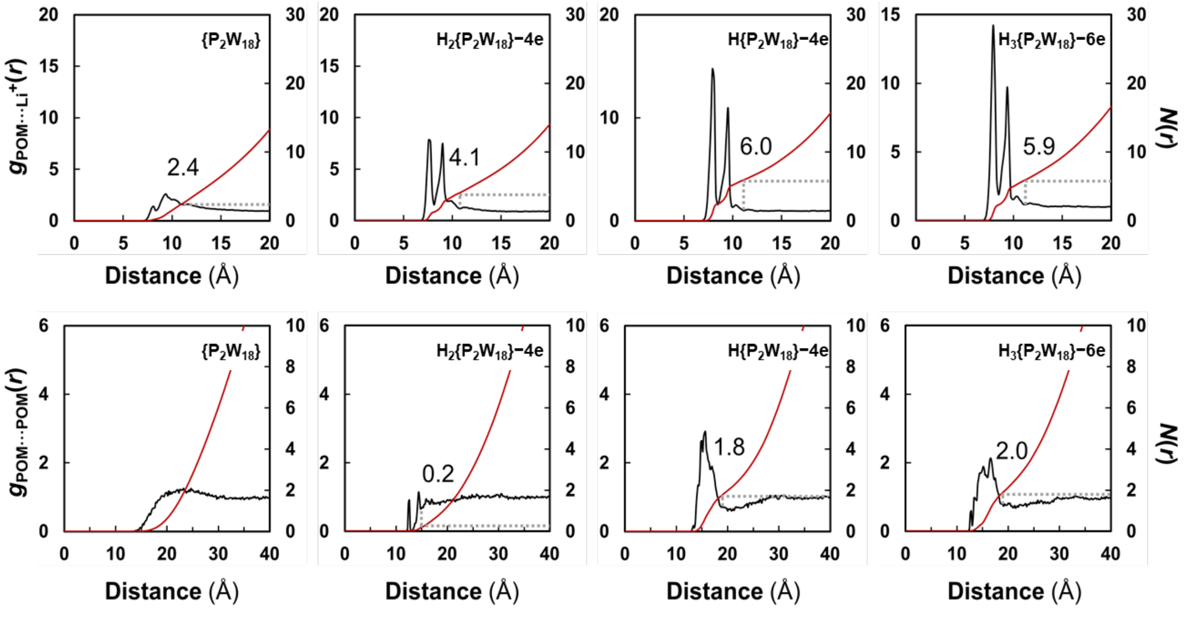

Figure S8-3: Compilation of the POM···POM and the POM···Li^+ RDFs computed taking as a reference the center of mass of both species. Data averaged over the last 10 ns of simulation sampling data every 2 ps. Black lines represent the RDF itself, $g(r)$, and red lines the coordination number, $N(r)$, obtained from integration of the RDF.

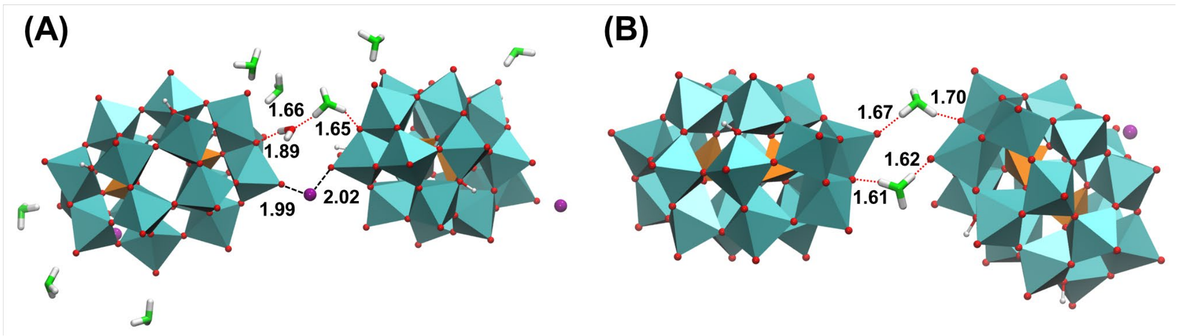

Figure S8-4: Representative snapshots of the interactions between **$H_3\{P_2W_{18}\}$−6e** anions. Li^+ are represented as purple spheres and H_3O^+ cations are highlighted in green. Snapshot (A) shows a lithium−mediated contact and another one involving a H_3O^+ cation and a water molecule (POM···POM distance from COMs, $r_{POM\cdots POM}$ = 16.0 Å) and (B) illustrates another interaction mode in which the POMs are linked by two H_3O^+ cations ($r_{POM\cdots POM}$ = 15.8 Å).

3. The W_{18}-18e species: structure and aggregation ability.

An exploratory study revealed that the aggregation ability of super-reduced clusters decreases with the number of associated protons; and that the agglomeration is more important when protons are accommodated in bridging oxygen atoms, as the terminal ones are more accessible to interact with the countercations. For this reason, we initially considered for the **$\{P_2W_{18}\}$−18e** anion the upper−limit case of having 18 protons in the structure. The stability of the super reduced species with 18 protons in bridging oxygens was evaluated by means of a DFT-MD run in water (see Figure S8-5). As the system is expected to have a lower number of protons attached, we simulated the system at neutral pH in a cubic simulation box of 25^3 Å, in such a way that the release of the first proton will already lead to a very low pH that reproduces the highly acidic experimental conditions. Analogously to the 4e−reduced POM, this *ab initio* simulation was carried out with spin polarization. As expected, the 18 protons−containing cluster releases a proton at the beginning of the simulation but after 1.2 ps the proton returns to the cluster at a terminal oxo site. The species with 18 protons (17 bridging, 1 terminal) lasts for *ca.* 1 ps and after that, another proton from a bridging position is released to the solution. No additional acid/base processes were observed during the remaining ~2 ps of simulation (see Figure S8-5), but at ~4.4 ps, another proton from a bridging position leaves to the bulk yielding the **$H_{16}\{P_2W_{18}\}$−18e** species, which remains until the end of the simulation. As initial approach, we performed the simulation with spin polarization constraining the spin multiplicity to 1, and the wave function evolved to an open−shell singlet configuration in which all the 18 electrons are unpaired and localized over the 18 W atoms.

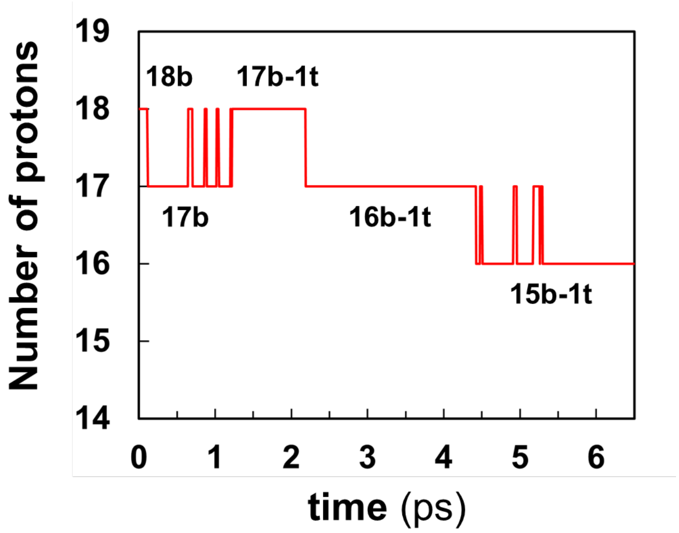

Figure S8-5: Evolution of the number of protons associated to **$\{P_2W_{18}\}$−18e** during a 6.5 ps DFT-MD simulation. A distance threshold of ≤1.3 Å was considered for a O—H bond. The labels "b" and "t" stand for protons at bridging and terminal oxygen atoms, respectively. Data were sampled every 7.2 fs.

The most abundant species in the simulation is the 17 proton−containing POM with 16 protons being at bridging positions. For this reason, we took a representative geometry for the **$H_{17}\{P_2W_{18}\}$−18e** anion from the trajectory to perform quantum mechanics calculations aiming to determine the electronic structure of the super−reduced species. In addition, DFT calculations discussed below suggested that the final structure is more likely to have 17 associated protons rather than 16 (*vide infra*). Optimization of the structure and the wave function locate one electron on each W center, combining population of d_{xy}−like orbitals with d_{xz} ones in the case of protonation at the terminal oxo group. These metal electrons were predicted to be unpaired but magnetically coupled to some extent, being the most likely configurations an open shell singlet followed by a quintet and a triplet states, which lie only 1.5 and 1.6 kcal·mol^{-1} above in energy (see Figure S8-6). Before reaching this conclusion we had to run dozens of calculations in an iterative process to evaluate the likelihood of several different electronic structures. For this reason, the accuracy of the calculations was limited to a double−ζ basis set. Even so, these calculations were capable of reproducing the qualitative trends required to rationalize the intricate super reduction process.

Figure S8-6A represents the spin density for the lowest in energy state, an open shell singlet, and Figure S8-6B shows a schematic MO diagram of the most likely configurations. Hence, this system is expected to possess a strong multi−configurational character although it might not show much intense paramagnetism, as suggested by EPR studies. Note that the B3LYP energy of the highest SOMO for **$H_{17}\{P_2W_{18}\}$−18e** with a bridging:terminal ratio of 16:1 is −3.12 eV, which is only *ca.* 1.40 V above the SOMO of **$\{P_2W_{18}\}$−1e** calculated with the same method. It is also worth to mention that a pure functional was used in the previous section to compare the energy levels of the structure with the experimental electrochemical data, as they give more realistic gaps between occupied and virtual orbitals compared to hybrid functionals, which tend to overstabilize occupied orbitals and in turn, to overestimate HOMO−LUMO gaps. However, a hybrid functional (B3LYP) was used for the comparison among electronic structures and molecular configurations because some percentage of HF exchange is usually required to efficiently localize electron density within a structure. This is of paramount importance when dealing with such large number of unpaired electrons. Otherwise, it is extremely difficult to achieve convergence in the optimization of the wave function during self−consistent field (SCF) iterations.

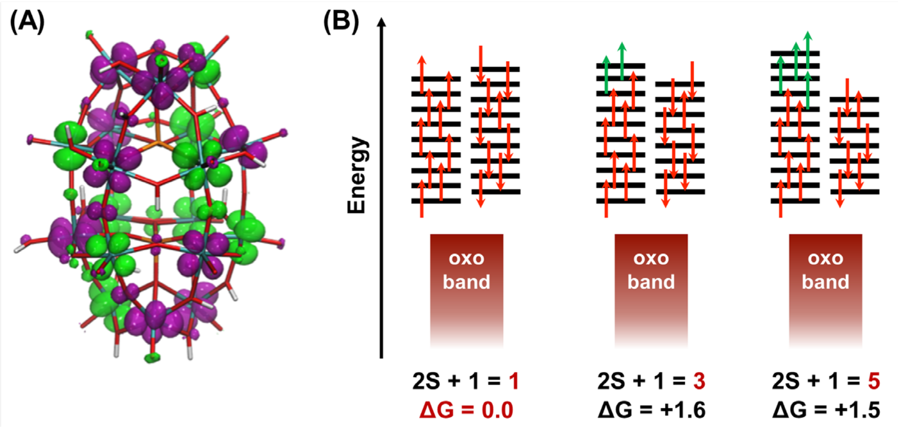

Figure S8-6. (A) Spin density representation for **$H_{17}\{P_2W_{18}\}$−18e** in an open−shell singlet configuration. Purple and green densities correspond to alpha and beta electrons, respectively. (B) Schematic MO diagram of the most likely electron configurations. Relative free energies were computed at B3LYP/LANL2DZ level. Electrons depicted in red and green denote "unpaired, magnetically-coupled" and "unpaired, non-coupled" electrons, respectively.

During the CPMD simulation described in Figure S8-5, a protonation event involving a terminal position was observed. To evaluate the proneness of the super reduced cluster to get protonated at terminal positions, we performed an additional static DFT study with the **$H_{17}\{P_2W_{18}\}$−18e** species moving protons from bridging to terminal oxygen atoms, and comparing their relative energy. Figure S8-7 exemplifies that moving three protons from bridging to terminal oxygen atoms stabilizes the super−reduced anion by 18.5 kcal·mol^{-1} in terms of free energy. In fully oxidized anions or when the number of electrons in the tungsten d band is small, there is a clear preference for protonating the bridging oxygens over terminal ones. Thus, the counterintuitive protonation at terminal oxygens in super reduced anions might be a consequence of the stabilization of a d_{xz} or a d_{yz} orbital below the highest SOMO upon lengthening the W—O distance with protonation at the terminal oxygen. This stabilization allows an electron to hop from the less stable d_{xy} orbital to a d_{yz}/d_{xz} that now is lower in energy, conferring stability to the reduced POM structure. This concept is graphically illustrated in Figure S8-8.

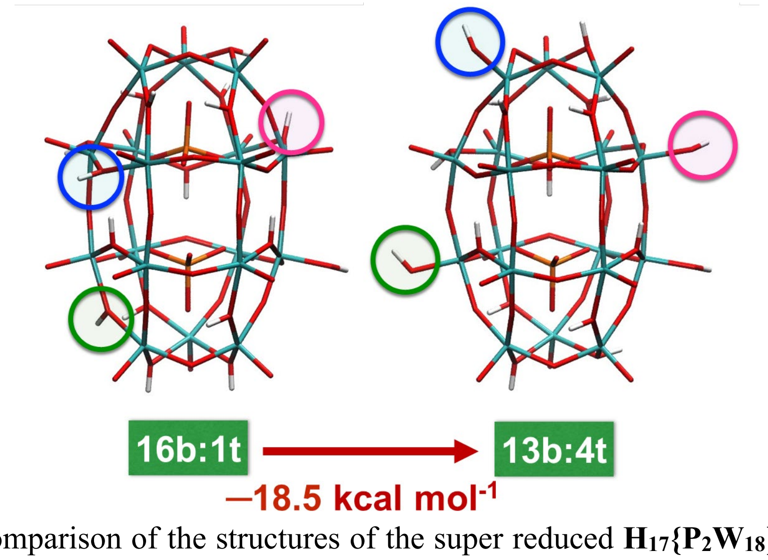

Figure S8-7: Comparison of the structures of the super reduced **$H_{17}\{P_2W_{18}\}$−18e** anion with bridging:terminal ratios of 16:1 and 13:4; highlighting the proton positions in which they differ from each other and the free energy change in going from 16:1 to 13:4.

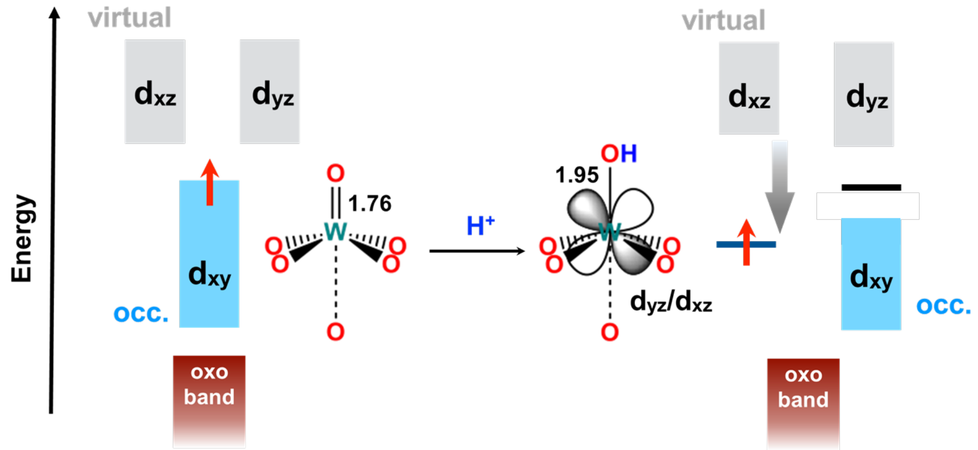

Figure S8-8: Schematic frontier orbital diagram illustrating the origin of stabilization upon protonating a terminal oxygen in highly reduced POMs.

Moving more protons in this structure might lead to artifacts in the quantification of the free−energy change as all the remaining protons participate in intramolecular hydrogen bonds. For this reason, we performed partial geometry optimizations to keep only one hydrogen bond in the optimized structure, which is the average number of intramolecular hydrogen bonds obtained from the CPMD simulation with explicit solvent. To do so, we constrained all the P···O—H angles but one to 160º to mimic the geometry that the hydroxo groups adopt in solution to form hydrogen bonds with solvent water molecules instead than with other oxygen atoms of the POM structure. This approach allowed a systematic analysis of different structures with different *bridging:terminal* ratios, all them with 17 protons (Table S8-2). Comparison of the relative free energies strongly suggests that the hyper−reduced cluster in aqueous solution might combine protons at bridging and terminal oxygen sites, the species with 7 protons at bridging positions and 10 at terminal ones (7:10) being the most likely proton distribution among the analyzed ones (see second column of Table S8-2**Table**). Besides, the 4:13 (+3.2 kcal·mol^{-1}), the 10:7 (+10.2 kcal·mol^{-1}) or the 13:4 (+14.2 kcal·mol^{-1}) ones and probably those presenting a proton distribution in between are also rather likely considering that most of the POMs in solution are not as isolated monomers but forming part of a supramolecular agglomerate, in which intermolecular interactions could alter their relative stabilities. Nevertheless, it is important to note that all of them can form agglomerates in a similar manner

as shown in Table S8-2 and Figure 4b of the main text and therefore, none of them is incompatible with the proposed mechanism for the super−reduction process.

Table S8-2: Main Features Concerning the Electronic Structure and Collective Behavior of **$H_{17}\{P_2W_{18}\}$−18e** with Different "*bridging:terminal*" Protonation Ratios.[a]

bridging: terminal	**ΔG**	**E_{SOMO}**	**RDF POM···POM**		**RDF POM···Li^+**		**RDF POM···H_3O^+**	
			N_{POM}	$r_{integration}$	N_{Li+}	$r_{integration}$	N_{H3O+}	$r_{integration}$
16:1	+32.7	–3.12	2.73	18.6	5.52	9.54	1.30	10.08
13:4	+14.2	–3.28	1.68	17.7	4.69	9.74	1.06	10.26
10:7	+10.2	–3.26	1.34	16.8	4.63	9.58	1.05	10.08
			0.06[b]	16.8[b]	2.13[b]	9.68[b]	0.71[b]	10.08[b]
**7:10**	**0.0**	**–3.52**	**2.88**	**17.1**	**7.06**	**9.72**	**1.19**	**9.90**
4:13	+3.2	–3.33	–	–	–	–	–	–
0:17	+30.5	–3.11	2.60	18.3	5.97	9.54	1.32	10.04

[a] All geometry optimizations were performed constraining the P···O_{bridge}H angles to 160º but one, according to the number of intramolecular hydrogen bonds in the CPMD simulation with explicit solvent. The selected phosphorus is that of the POM hemisphere in which the hydroxo group is found. No constrains were used for the 0:17 isomer as it does not contain any proton at a bridging position. Relative Free energies are given in kcal·mol^{-1} and orbital energies in eV. The integration distances for both RDFs are given in Å and refer to the centers of mass of both species. [b] Simulation at a POM concentration of 20 mM.

Similarly to what we observed for **H$\{P_2W_{18}\}$−4e** in Figure 3c, the agglomeration of **$H_{17}\{P_2W_{18}\}$−18e (7b:10t)** anions was found to lower the energy of their highest SOMO by 250 mV. Notably, the energy of the highest SOMO of **$H_{17}\{P_2W_{18}\}$−18e (7b:10t)** in the agglomerate represented in Figure S8-9 (−3.78 eV; B3LYP/LANL2DZ and continuum solvent) is only 0.76 eV higher in energy than the SOMO of the 1e−reduced **$\{P_2W_{18}\}$−1e** (−4.54 eV) computed at the same level of theory, what fully agrees with the observed voltage *window of 0.8 V* for the re−oxidation process.[1] It is also worth mentioning that the spin density in the structure of the

agglomerate is also localized mainly on W atoms (Figure S8-9b). Overall, these results clearly show that without the additional stabilization provided by the enhanced ion pairing upon agglomeration, the formation of the hyper-reduced anions would not be possible as the SOMOs in highly reduced species would be too high in energy with respect to those in the initial redox states. Further supporting this conclusion, the **$H_{17}\{P_2W_{18}\}$−18e (7b:10t)** anion showed almost no agglomeration at a concentration of 20 mM (see Table S8-2 and Figure 4e). Therefore, agglomeration is not expected neither at a 10−fold lower concentration (2 mM), in agreement with the experimental impossibility of reducing **$\{P_2W_{18}\}$** beyond six electrons at these conditions.

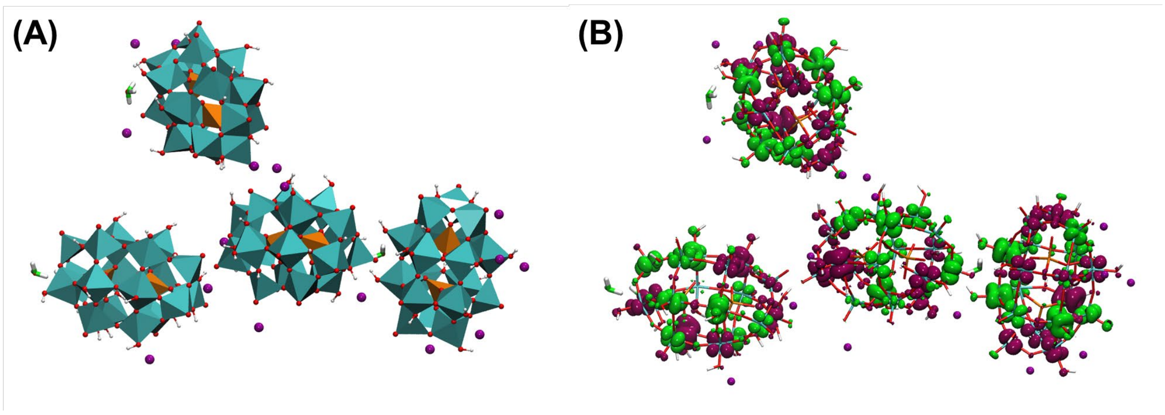

Figure S8-9: (A) Snapshot of the agglomerate fragment used to evaluate the stabilization in the MOs of the hyper-reduced anion **$H_{17}\{P_2W_{18}\}$−18e (7b:10t)** caused by agglomeration. Li^+ cations are represented as purple spheres and H_3O^+ cations are highlighted in green. (B) Spin density of the agglomerate structure with 72 unpaired electrons, computed as open−shell singlet.

To evaluate the stability of the **$H_{17}\{P_2W_{18}\}$−18e (7b:10t)** anion, we performed an additional 10 ps DFT-MD simulation of this anion in water at pH 7 that showed that the anion keeps the 17 protons during the 99.6 % of the simulated time. Therefore, it is assumable that **$H_{17}\{P_2W_{18}\}$−18e (7b:10t)** represents a realistic molecular model of the super−reduced POM in solution. Furthermore, static DFT calculations indicate that abstracting one proton from this species is endergonic by 5.9 kcal·mol^{-1} (Table S8-3) and thus, the **$H_{16}W_{18}$−18e(15:1)** species

observed in the DFT-MD starting from the **$H_{18}W_{18}$−18e** anion might be an intermediate in the formation of a more stable structure, such as the **$H_{17}\{P_2W_{18}\}$−18e (7b:10t)** one, which is more than 30 kcal·mol^{-1} more stable (see Table S8-2). Besides, the highest SOMO in **$H_{16}\{P_2W_{18}\}$−18e (7b:9t)** is almost 200 mV above than in **$H_{17}\{P_2W_{18}\}$−18e (7b:10t)**, what is not expected to be compensated with the increase of ion pairing and agglomeration upon increasing the negative charge of the cluster from 7– to 8–. Observing the complete transformation of **$H_{17}\{P_2W_{18}\}$−18e (16b:1t)** into **$H_{17}\{P_2W_{18}\}$−18e (7b:10t)** by means of DFT-MD simulations would require much longer simulation time, as it may involve a vast number of proton transfer steps. On the other hand, Table S8-3 shows that protonating a terminal position in **$H_{17}\{P_2W_{18}\}$−18e (7b:10t)** is exergonic by 6.0 kcal·mol^{-1} and goes along with a little stabilization of the highest SOMO. Based on these results, it is reasonable to conclude that hyper-reduced anions should have a number of associated protons equal to 17 or 18, as the stabilization or destabilization induced by protonation or deprotonation might be compensated by the change in the agglomeration ability in the presence of alkali cations, which increases with the POM charge.

Table S8-3: Comparison between several **$\{P_2W_{18}\}$−18e** structures with different protonation states and patterns.[a]

anion	*n* H^+	*q*	*n* unpaired e^-	E_{SOMO}	ΔG
**$H_{17}\{P_2W_{18}\}$−18e (7b:10t)**	17	7–	18	–3.52	0.0
**$H_{16}\{P_2W_{18}\}$−18e (7b:9t)**	16	8–	18	–3.35	+5.9
**$H_{18}\{P_2W_{18}\}$−18e (7b:11t)**	18	6–	18	–3.59	−6.0
**$H_{17}\{P_2W_{18}\}$−18e (6b:7t:2*aqua*)**	17	7–	16	–3.30	+13.6
**$H_{17}\{P_2W_{18}\}$−18e (3b:2t:6*aqua*)**	17	7–	6	–3.31	+18.6

[a]All geometry optimizations were performed constraining the P···O_{bridge}H angles to 160º but one, according to the number of intramolecular hydrogen bonds in the DFT-MD simulation with explicit solvent. The selected phosphorus is that of the POM hemisphere in which the hydroxo group is found. The energy of the highest SOMO is given in eV and relative Gibbs free energies in kcal·mol^{-1}.

Previous electrochemical studies suggested that the six−electron−reduced metatungstate $[H_nW_{12}O_{40}]^{(14-n)-}$ exhibits three W—W bonds within a triad that gather all the extra electrons.[4,5] In addition, the formation of the W—W bonds was proposed to be coupled to the transformation of the terminal oxo ligands into more labile aqua ligands.[5] To explore this possibility, we computed two additional structures containing 1 and 6 W—W bonds at the cap regions, labeled as **$H_{17}\{P_2W_{18}\}$−18e (6b:7t:2*aqua*)** and **$H_{17}\{P_2W_{18}\}$−18e (3b:2t:6*aqua*)**, respectively. As depicted in Figure S8-10, the optimized structure of **$H_{17}\{P_2W_{18}\}$−18e (3b:2t:6*aqua*)** displays 3 W^{IV} ions in each cap, linked to each other by metallic bonds that range from 2.62 to 2.65 Å. The belt regions are mixed−valent, having W ions with oxidation states of 5+ and 6+. On the other hand, **$H_{17}\{P_2W_{18}\}$−18e (6b:7t:2*aqua*)** is more anisotropic, as only two W^{IV} ions are found in one cap and the remaining 14 electrons are delocalized over the remaining W atoms. In these structures, the overall number of protons was maintained as 17, what allowed us to compare their relative stability to the **$H_{17}\{P_2W_{18}\}$−18e (7b:10t)**. The last two entries in Table S8-3 show that the formation of W—W bonds (along with the electron−electron pairing) does not stabilize the structure of the hyper−reduced anions, which prefer to keep unpaired all the *d* electrons. In fact, the formation of a single metallic bond is destabilizing by 13.6 kcal·mol^{-1} and it causes the energy of the highest SOMO to be shifted up by ca. 200 mV with respect to the all−W^V structure (Table S8-3). Thus, unlike other reduced W−based structures, the 18−electron reduced Wells−Dawson does not seem likely to form W—W bonds. Furthermore, this is supported by the fact that the experimental reduction of **$\{P_2W_{18}\}$** implicates only 18 electrons, because if the formation of W—W bonds were possible, further reduction of the cluster beyond 18 electrons might be observed owing the presence of fully oxidized W^{VI} ions in the structure.

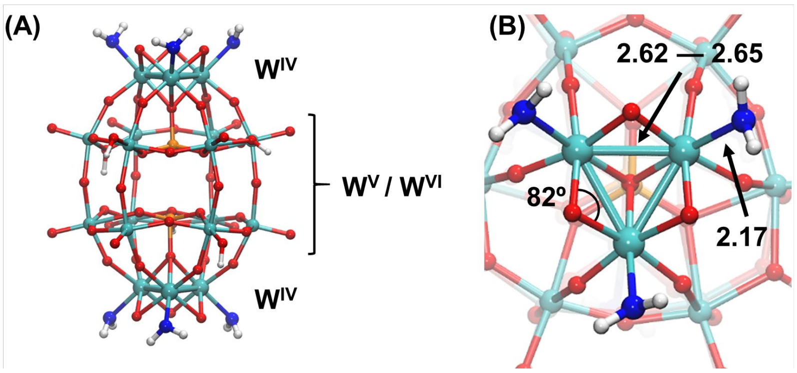

Figure S8-10: (A) Optimized structure of **$H_{17}\{P_2W_{18}\}$−18e (3b:2t:6*aqua*)** species presenting 6 W^{IV}—W^{IV} bonds in the cap regions and W^V/W^{VI} mixed−valent belts. Aqua ligands are colored in blue. (B) Top view of the same cluster in which main geometrical parameters are given in Å and degrees (º).

References SI-8

1 Kelly, C. P.; Cramer, C. J.; Thrular, D. G. *J. Phys. Chem. B* **2007**, *111*, 408-422.

2 López, X.; Carbó, J. J.; Bo, C.; Poblet, J. M. *Chem. Soc. Rev.* **2012**, *41*, 7537-7571.

3 Solé-Daura, A.; Notario-Estévez, A.; Carbó, J. J.; Poblet, J. M.; De Graaf, C.; Monakhov, K. Y.; López, X. *Inorg. Chem.* **2019**, *58*, 3881–3894.

4 Launay, J. P. *J. Inorg. Nucl. Chem.* **1976**, *38*, 807−816.

5 a) Jeannin, Y.; Launay, J. P.; Seid Sedjadi, M. A. *Inorg. Chem.* **1980**, *19*, 2933−2935; b) Piepgrass, K.; Pope, M. T. *J. Am. Chem. Soc.* **1987**, *109*, 1586−1587; c) Boskovic, C.; Sadek, M.; Brownlee, R. T. C.; Bond, A. M.; Wedd, A. G. *J. Chem. Soc., Dalton Trans.* **2001**, *2001*, 187−196

SI-9 Computational Details.

1. Static DFT calculations

To determine redox potentials, we initially optimized the structures at BP86[1] level of theory with a Slater−type TZP basis set using the ADF2016.01 code.[2] Solvent effects of water were taken into account with the COSMO[3] continuum solvent model (ε = 78.4) and relativistic effects were considered by means of the ZORA approximation.[4] The remaining calculations were performed with Gaussian09 (rev D.01)[5] using the hybrid B3LYP functional[6] and a LANL2DZ basis set[7] for all atoms, with the corresponding pseudopotential for W and P centers. In this case, solvent effects were simulated by means of the IEF−PCM solvent model[8] and frequency calculations were performed at 298 K and 1 atm. To evaluate protonation energies, we used the experimental value of solvation free energy of a proton in solution[9] and the free−energy change in (de)protonation processes was corrected with the standard state correction of 1.9 $kcal \cdot mol^{-1}$ that accounts for the change in free energy in going from gas phase at 1 atm to the standard state of 1 M in solution at 298 K.

2. Classical MD simulations.

The systems were simulated by classical MD using the GROMACS 4.5.4 software[10] and the AMBER99 force field.[11] Force field parameters for POMs were obtained following the procedure of Bonet−Avalos, Bo, Poblet et al,[12] using CHELPG atomic charges derived from the electrostatic potential computed in the gas-phase. They were obtained with the Gaussian09 package[5] at the DFT level (BP86 functional)[1] using the LANL2DZ basis set[7] Solvent effects were included in geometry optimizations by using the IEF−PCM model[8] as implemented in Gaussian09.[5] The set of Lennard−Jones parameters for W and O atoms were taken from previous work,[13] Water was represented with the TIP3P model.[14] All simulations were performed with 3D−periodic boundary conditions using an atom cutoff of 14 Å for 1−4 van

der Waals and of 10 Å for 1−4 Coulombic interactions and corrected for long−range electrostatics by using the particle−particle mesh Ewald (PME) summation method.[15] The simulations were performed at 300 K starting with random velocities. The temperature was controlled by coupling the system to a thermal bath using the Velocity-rescaling algorithm[16] with a relaxation time of 0.5 ps to keep the NVT canonical conditions throughout the simulation. Newton equations of motion were integrated using the leap−frog algorithm,[17] and a time step of 1 fs. The bonds with hydrogens were restrained using the LINCS algorithm.[18] Before the production runs, the systems were equilibrated with 5000 steps of energy minimization followed by simulations of 250 ps at constant volume (NVT), 500 ps at NPT conditions and a final equilibration of 250 ps under NVT. Finally, all systems were simulated for 40 ns under NVT conditions.

In all cases, 50 POM anions were embedded in a cubic solvent box of 94.0^3 Å^3, as well as 300 Li^+ and the number of H_3O^+ required to neutralize the charge of the system. Force field parameters for hydronium cations were taken from the AMBER99[11] force field and the TIP3P water model.[14] Equilibrium distances and angles were taken from the DFT−optimized geometry, obtained at BP86[1] level with a 6−311G(d,p) basis set[19] for all atoms using Gaussian09[5] and the IEF−PCM[8] continuum solvent model (ε = 78.4). Atomic charges were computed from a single−point calculation in gas−phase[12] using the same level of theory, as done for all the simulated POM structures. This procedure was already followed by Wippf and co−workers to model the agglomeration of polyoxotungstates with H_3O^+ cations as linkers.[20] Simulations at lower POM concentration (20 mM) were performed in a simulation box of the same dimensions but decreasing the number of POMs from 50 to 10 and the number of counter cations, accordingly.

3. DFT-MD simulations

DFT-MD simulations were using the CPMD 4.1 code,[21] and adopting the generalized gradient−corrected BLYP exchange−correlation functional.[22] The electronic structure was described by expansion of the valence electronic wave functions into a plane−wave basis set, which is limited by an energy cut−off of 70 Ry. The interaction between the valence electrons and the ionic cores was treated using the pseudopotential (PP) approximation. Norm−conserving Troullier−Martins PPs were used for W, P, O and Li centers,[23] which nonlocal part was calculated using the Kleinman−Bylander method.[24] H atoms were described with a Goedecker−type PP.[25] During the MD simulations, the wave functions are propagated in the Car−Parrinello scheme, by integrating the equations of motion derived from the Car−Parrinello Lagrangian.[26] A fictitious electronic mass of 900 au was employed with a time step of 0.144 fs, as H atoms were substituted by D. The Nosé−Hoover thermostat[27] for the nuclear degrees of freedom was used to maintain a temperature at 300 K. All simulations were performed with spin polarization and with 3D-periodic boundary conditions starting from geometries that were previously equilibrated with classical MD.

Three main simulations are discussed in the text: they all contain one POM cluster embedded in periodic cells but of different size. The first simulation was performed in a cubic cell of 25^3 Å^3 to avoid possible interactions between POM clusters in neighboring boxes upon rotation of the cluster from its initial position, as Wells−Dawson anions have $x = y \neq z$ dimensions. Nevertheless, as this rotation was not observed at the simulation time scale, further simulations were performed with orthorhombic cells of 20 × 20 × 25 Å^3 (with $\alpha = \beta = \gamma = 90^o$) to reduce their computational cost. Thus, the simulated systems are:

- **{P_2W_{18}}−4e** in acid medium: one **{P_2W_{18}}−4e** anion (q = 10−) embedded in a box of 20 × 20 × 25 Å^3 with 6 H_3O^+ cations, 4 Li^+ cations and 233 water molecules (935 atoms). The system was simulated for 5.5 ps.

- **$H_{18}\{P_2W_{18}\}$−18e** at neutral pH: one **$H_{18}\{P_2W_{18}\}$−18e** anion (*q* = 6−) in a 25^3 Å^3 box with 6 Li^+ cations and 428 water molecules (1390 atoms). The system was simulated for 6.5 ps.
- **$H_{17}\{P_2W_{18}\}$−18e**(7:10) at neutral pH: one **$H_{17}\{P_2W_{18}\}$−18e** (7:10) anion (*q* = 7−) in a 20 × 20 × 25 Å^3 box with 4 Li^+ cations and 265 water molecules (898 atoms). This simulation was carried out for 10 ps.

In the simulation with **$H_{18}\{P_2W_{18}\}$−18e**, a number of cations to neutralize the overall charge was included in the periodic box, while in other simulations, the number of cations corresponds to the average coordination numbers within 20 Å from the POM center of mass computed from 5 ns classical MD. Previous classical simulations were performed using 6 Li^+ because of the 6:1 stoichiometry of the initial POM salt and when simulating low pH, the number of H_3O^+ ions to reach a concentration equivalent to a pH < 1 was added in the box.

References SI-9

1. a) Perdew, J. P. Phys. Rev. B: Condens. Matter Mater. Phys. **1986**, 33, 8822−8824; b) Perdew, J. P. Phys. Rev. B: Condens. Matter Mater. Phys. **1986**, 34, 7406; c) Becke, A. D. Phys. Rev. A: At., Mol., Opt. Phys. **1988**, 38, 3098−3100.
2. a) Te Velde, G.; Bickelhaupt, F. M.; van Gisbergen, S. J. A.; Fonseca Guerra, C.; Baerends, E. J.; Snijders, J. G.; Ziegler, T. *J. Comput. Chem.* **2001**, *22*, 931−967; b) Fonseca Guerra, C.; Snijders, J. G.; Te Velde, G.; Baerends, E. J. *Theor. Chem. Acc.* **1998**, *99*, 391−403; c) ADF2016, SCM, Theoretical Chemistry; Vrije Universiteit: Amsterdam, The Netherlands, Http://Www.Scm.Com.
3. a) Klamt, A. *J. Phys. Chem.* **1995**, *99*, 2224−2235; b) Klamt, A.; Jonas, V.; Bürger, T.; Lohrenz, J. C. W. *J. Phys. Chem. A* **1998**, *102*, 5074−5085; c) Pye, C. C.; Ziegler, T. *Theor. Chem. Acc.* **1999**, *101*, 396−408.

4. a) van Lenthe, E.; Baerends, E. J.; Snijders, J. G. *J. Chem. Phys.* **1993**, *99*, 4597−4610; b) van Lenthe, E.; Baerends, E. J.; Snijders, J. G. *J. Chem. Phys.* **1994**, *101*, 9783−9792; c) Van Lenthe, E.; Ehlers, A.; Baerends, E. J. *J. Chem. Phys.* **1999**, *110*, 8943−8953; d) Klopper, W.; van Lenthe, J. H.; Hennum, A. C. *J. Chem. Phys.* **2000**, *113*, 9957−9965.
5. Frisch, M. J.; Trucks, G. W.; Schlegel, H. B.; Scuseria, G. E.; Robb, M. A.; Cheeseman, J. R.; Scalmani, G.; Barone, V.; Mennucci, B.; Petersson, G. A.; Nakatsuji, H.; Caricato, M.; Li, X.; Hratchian, H. P.; Izmaylov, A. F.; Bloino, J.; Zheng, G.; Sonnenberg, J. L.; Hada, M.; Ehara, M.; Toyota, K.; Fukuda, R.; Hasegawa, J.; Ishida, M.; Nakajima, T.; Honda, Y.; Kitao, O.; Nakai, H.; Vreven, T.; Montgomery, J. A., Jr.; Peralta, J. E.; Ogliaro, F.; Bearpark, M.; Heyd, J. J.; Brothers, E.; Kudin, K. N.; Staroverov, V. N.; Kobayashi, R.; Normand, J.; Raghavachari, K.; Rendell, A.; Burant, J. C.; Iyengar, S. S.; Tomasi, J.; Cossi, M.; Rega, N.; Millam, J. M.; Klene, M.; Knox, J. E.; Cross, J. B.; Bakken, V.; Adamo, C.; Jaramillo, J.; Gomperts, R.; Stratmann, R. E.; Yazyev, O.; Austin, A. J.; Cammi, R.; Pomelli, C.; Ochterski, J. W.; Martin, R. L.; Morokuma, K.; Zakrzewski, V. G.; Voth, G. A.; Salvador, P.; Dannenberg, J. J.; Dapprich, S.; Daniels, A. D.; Farkas, Ö.; Foresman, J. B.; Ortiz, J. V.; Cioslowski, J.; Fox, D. J. Gaussian 09, revision D.01; Gaussian, Inc.: Wallingford, CT, 2009.
6. a) Lee, C.; Yang, W.; Parr, R. G. *Phys. Rev. B: Condens. Matter Mater. Phys.* **1988**, *37*, 785−789; b) Becke, A. D. *J. Chem. Phys.* **1993**, *98*, 5648−5652; c) Stephens, P. J.; Devlin, F. J.; Chabalowski, C. F.; Frisch, M. J. *J. Phys. Chem.* **1994**, *98*, 11623−11627.
7. Hay, P. J.; Wadt, W. R. *J. Chem. Phys.* **1985**, *82*, 270−283.
8. Cancès, E.; Mennucci, B.; Tomasi, J. *J. Chem. Phys.* **1997**, *107*, 3032−3041.
9. Tissandier, M. D.; Cowen, K. A.; Feng, W. Y.; Gundlach, E.; Cohen, M. H.; Earhart, A. D.; Coe, J. V.; Tuttle, T. R. *J. Phys. Chem. A* **1998**, *102*, 7787−7794.

10. a) Hess, B.; Kutzner, C.; Van Der Spoel, D.; Lindahl, E. *J. Chem. Theory Comput.* **2008**, *4*, 435−447; b) Van Der Spoel, D.; Lindahl, E.; Hess, B.; Groenhof, G.; Mark, A. E.; Berendsen, H. J. C. *J. Comput. Chem.* **2005**, *26*, 1701−1718; c) Berendsen, H. J. C.; Van Der Spoel, D.; van Drunen, R. *Comput. Phys. Commun.* **1995**, *91*, 43−56.
11. Wang, J. M.; Cieplak, P.; Kollman, P. A. *J. Comput. Chem.* **2000**, *21*, 1049−1074.
12. a) Chaumont, A.; Wipff, G. *Phys. Chem. Chem. Phys.* **2008**, *10*, 6940−6953; b) Chaumont, A.; Wipff, G. *J. Phys. Chem. C* **2009**, *113*, 18233−18243; c) Chaumont, A.; Wipff, G. *C. R. Chimie*, **2012**, *15*, 107−117.
13. a) Leroy, F.; Miró, P.; Poblet, J. M.; Bo, C.; Bonet Ávalos, J. *J. Phys. Chem. B* **2008**, *112*, 8591−8599; b) López, X.; Nieto−Draghi, C.; Bo, C.; Avalos, J. B.; Poblet, J. M. *J. Phys. Chem. A* **2005**, *109*, 1216−1222.
14. Jorgensen, W. L.; Chandrasekhar, J.; Madura, J. D.; Impey, R. W.; Klein, M. L. *J. Chem. Phys.* **1983**, *79*, 926−935.
15. Darden, T.; York D.; Pedersen, L. *J. Chem. Phys.* **1993**, *98*, 10089−10092.
16. Bussi, G.; Donadio, D.; Parrinello, M. *J. Chem. Phys.* **2007**, *126*, 014101.
17. Hockney, R. W.; Goel, S. P.; Eastwood, J.; Quiet, J. *J. Comp. Phys.* **1974**, *14*, 148–158.
18. Hess, B.; Bekker, H.; Berendsen, H. J. C.; Fraaije, J. G. E. M. *J. Comp. Chem.* **1997**, *18*, 1463–1472.
19. Krishnan, R.; Binkley, J. S.; Seeger, R.; Pople, J. A. *J. Chem. Phys.* **1980**, *72*, 650−654.
20. Chaumont, A.; Wipff G. *Phys. Chem. Chem. Phys.* **2008**, *10*, 6940−6953.
21. IBM Corp.Armonk, N. Y. CPMD; MPI für Festkörperforschung: Stuttgart, Germany, 1990−2006; pp 1997−2001.
22. a) Lee, C.; Yang, C.; Parr, R. G. *Phys. Rev. B: Condens. Matter Mater. Phys.* **1988**, *37*, 785−789; b) Becke, A. D. *Phys. Rev. A: At., Mol., Opt. Phys.* **1988**, *38*, 3098−3100.

23. Troullier, N.; Martins, J. L. *Phys. Rev. B: Condens. Matter Mater. Phys.* **1991**, *43*, 1993−2006.

24. Kleinman L.; Bylander, D. M. *Phys. Rev. Lett.* 1982, 48, 14251428.

25. Hartwigsen, C.; Goedecker, S.; Hutter J. *Phys. Rev. B* **1998**, *58*, 3641.

26. Car, R.; Parrinello, M. *Phys. Rev. Lett.* **1985**, *55*, 2471−2474.

27. a) Nosé, S. *J. Chem. Phys.* **1984**, *81*, 511−519; b) Hoover, W. G. *Phys. Rev. A: At., Mol. Opt. Phys.* **1985**, *31*, 1695−1697.
